# Acute kidney injury in patients with COVID-19 compared to those with influenza: a systematic review and meta-analysis

**DOI:** 10.3389/fmed.2023.1252990

**Published:** 2023-09-19

**Authors:** Chiu-Ying Hsiao, Heng-Chih Pan, Vin-Cent Wu, Ching-Chun Su, Tzu-Hsuan Yeh, Min-Hsiang Chuang, Kuan-Chieh Tu, Hsien-Yi Wang, Wei-Chih Kan, Chun-Chi Yang, Jui-Yi Chen

**Affiliations:** ^1^Division of Nephrology, Department of Internal Medicine, Chi-Mei Medical Center, Tainan, Taiwan; ^2^Graduate Institute of Clinical Medicine, College of Medicine, National Taiwan University, Taipei, Taiwan; ^3^Division of Nephrology, Department of Internal Medicine, Keelung Chang Gung Memorial Hospital, Keelungi, Taiwan; ^4^Chang Gung University College of Medicine, Taoyuan, Taiwan; ^5^Community Medicine Research Center, Keelung Chang Gung Memorial Hospital, Keelung, Taiwan; ^6^Department of Internal Medicine, National Taiwan University Hospital, Taipei, Taiwan; ^7^Division of Cardiology, Department of Internal Medicine, Chi Mei Medical Center, Tainan, Taiwan; ^8^Department of Sport Management, College of Leisure and Recreation Management, Chia Nan University of Pharmacy and Science, Tainan, Taiwan; ^9^Department of Medical Laboratory Science and Biotechnology, Chung Hwa University of Medical Technology, Tainan, Taiwan; ^10^Division of Hepato-gastroenterology, Department of Internal Medicine, Chi Mei Medical Center, Tainan, Taiwan; ^11^Department of Health and Nutrition, Chia Nan University of Pharmacy and Science, Tainan, Taiwan

**Keywords:** COVID-19, influenza, acute kidney injury, recovery, mortality

## Abstract

**Background:**

COVID-19 and influenza can both lead to acute kidney injury (AKI) as a common complication. However, no meta-analysis has been conducted to directly compare the incidence of AKI between hospitalized patients with COVID-19 and influenza. The objective of our study aims to investigate the incidence and outcomes of AKI among hospitalized patients between these two groups.

**Materials and methods:**

A systematic search of PubMed, Embase, and Cochrane databases was conducted from December 2019 to August 2023 to identify studies examining AKI and clinical outcomes among hospitalized patients with COVID-19 and influenza. The primary outcome of interest was the incidence of AKI, while secondary outcomes included in-hospital mortality, recovery from AKI, hospital and ICU stay duration. The quality of evidence was evaluated using Cochrane and GRADE methods.

**Results:**

Twelve retrospective cohort studies, involving 17,618 hospitalized patients with COVID-19 and influenza, were analyzed. COVID-19 patients showed higher AKI incidence (29.37% vs. 20.98%, OR: 1.67, 95% CI 1.56–1.80, *p* < 0.01, I^2^ = 92.42%), and in-hospital mortality (30.95% vs. 5.51%, OR: 8.16, 95% CI 6.17–10.80, *p* < 0.01, I^2^ = 84.92%) compared to influenza patients with AKI. Recovery from AKI was lower in COVID-19 patients (57.02% vs., 80.23%, OR: 0.33, 95% CI 0.27–0.40, *p* < 0.01, I^2^ = 85.17%). COVID-19 patients also had a longer hospital stay (SMD: 0.69, 95% CI 0.65–0.72, *p* < 0.01, I^2^ = 98.94%) and longer ICU stay (SMD: 0.61, 95% CI 0.50–0.73, *p* < 0.01, I^2^ = 94.80%) than influenza patients. In our study, evidence quality was high (NOS score 7–9), with low certainty for AKI incidence and moderate certainty for recovery form AKI by GRADE assessment.

**Conclusion:**

COVID-19 patients had higher risk of developing AKI, experiencing in-hospital mortality, and enduring prolonged hospital/ICU stays in comparison to influenza patients. Additionally, the likelihood of AKI recovery was lower among COVID-19 patients.

## 1. Introduction

The COVID-19 pandemic is caused by severe acute respiratory syndrome coronavirus 2 (SARS-CoV-2), an exceptionally contagious virus that originated in Wuhan, China in late 2019. In February 2020, the World Health Organization (WHO) formally named the disease caused by SARS-CoV-2 as coronavirus disease 2019, or COVID-19 (1). As of 13 August 2023, the global count of confirmed cases has surpassed 769 million, with reported fatalities exceeding 6.9 million (2). COVID-19 primarily manifests as a respiratory disease, with its initial impact concentrated on the lungs. Early symptoms include fever, cough, and breathing difficulties. Nevertheless, COVID-19 patients have exhibited extrapulmonary involvement, affecting various regions including the heart, gastrointestinal tract, blood vessels, nervous system, and kidneys (3). Influenza is the most prevalent infectious disease compared to COVID-19 owing to its analogous transmission routes, clinical manifestation, and associated complications. Four types of seasonal influenza viruses exist: types A, B, C, and D. Among these, types A and B are clinically significant for humans and are responsible for generating seasonal epidemics. The Influenza A virus comprises the subtypes A(H1N1) and A(H3N2), both of which frequently circulate within in human populations. Notably, influenza type A viruses have been responsible for pandemics induced severe illness, particularly among older individuals and those with chronic conditions (4).

Growing evidence suggests that acute kidney injury (AKI) is a frequent complication of COVID-19, with older age, diabetes, hypertension, and chronic kidney disease (CKD) being major risk factors for its occurrence (5). The reported incidence of AKI in recent meta-analysis studies has shown a ranged between 8.9% and 75% in hospitalized patients with COVID-19 infection (6, 7). Moreover, 52% of COVID-19 patients with AKI have been reported to be critically ill (8), and to commonly require renal replacement therapy (RRT) (9). Additionally, it has been observed that hospitalized COVID-19 patients who develop AKI have been reported to exhibit a significantly elevated risk of mortality compared to those without AKI. Conversely the mortality rate in COVID-19 patients without AKI has been reported to be low (10). Similarly, influenza also has potential for pandemics, and the reported incidence of AKI in patients with influenza has been reported to a range from 17% ~ 61% (11–13). In addition, the necessity for RRT is also frequently observed for patients with influenza infection and AKI (14).

A recent meta-analysis (8) indicated that there were no significant differences in the frequencies of AKI between critically ill patients with COVID-19 and those with other respiratory viruses, including influenza. This study encompassed both ACE2-associated viruses (including influenza H1N1) and non-ACE2-associated viruses (other types of influenza). Nevertheless, the number of non-ACE2-associated groups was limited. Only a few studies have directly compared the incidence of AKI between patients with COVID-19 and influenza. Therefore, the aim of this meta-analysis was to assess and compare the incidence of AKI and kidney-related outcomes among patients with COVID-19 and those with influenza infection.

## 2. Methods

### 2.1. Literature search and study selection

We conducted an extensive literature search on PubMed, Embase, and Cochrane spanning from December 2019 to August 2023. No restrictions were applied to language or geographical location. The aim was to identify studies comparing the risks of AKI and clinical outcomes between patients with COVID-19 and influenza. Our search strategy employed a wide range of search terms, including “COVID-19,” “SARS-CoV-2,” “severe acute respiratory syndrome coronavirus 2,” “influenza,” “H1N1,” “H3N2,” “acute kidney injury,” “acute renal failure,” and “acute renal insufficiency.” Supplementary to the search, we manually scrutinized reference lists of relevant studies, systematic reviews, and meta-analyses to identify any additional pertinent publications for our analysis. Two authors (CC Hsiao; CC Su) independently evaluated the titles and abstracts of the identified articles to ascertain their eligibility for the final analysis. Subsequently, full-text papers were assessed for quality and data extraction. Our meta-analysis followed the guidelines outlined in the Preferred Reporting Items of Systematic Reviews and Meta-Analyses (PRISMA) statement (15) and Cochrane methods (16). The systematic review protocol was pre-established and registered on PROSPERO prior to commencement (17).

### 2.2. Inclusion and exclusion criteria

The included studies were all cohort studies which met the following eligibility criteria: (1) enrolled adults >18 years old; with (2) exposed population: hospitalized patients with COVID-19 infection; (3) control group: hospitalized patients with influenza infection; and (4) outcomes: incidence of AKI, in-hospital mortality with AKI, recovery from AKI, the length of hospitalization/intensive care unit (ICU) stay. Studies were excluded if they: (1) enrolled individuals <18 years old; (2) lacked data about the incidence of AKI; (3) were reviews, letters, conference abstracts, case reports, or studies other than original investigations; and (4) enrolled patients with prior dialysis before admission.

### 2.3. Data extraction and risk of bias assessment

Two investigators, CY Hsiao and CC Su meticulously extracted all pertinent data from the included studies. The characteristics of each study were documented, including the authors, publication year, geographic location of the study, study design, sample size, age distribution, gender distribution, types of influenza, and prevalent comorbid conditions (such as type 2 DM, hypertension, or CKD). Additionally, details of complications or outcomes were also captured, including the incidence of AKI, in-hospital mortality with AKI, recovery from AKI, occurrence of adult respiratory distress syndrome (ARDS), admission rate to an ICU, administration of ventilator therapy, utilization of extracorporeal membrane oxygenation (ECMO), duration of hospitalization or ICU stay. The primary outcome of this meta-analysis was the incidence of AKI, while the secondary outcomes encompassed in-hospital mortality with AKI, recovery from AKI, the length of hospitalization/ICU stay, and the rates of RRT, vasopressor therapy, ventilator therapy and ARDS. The criteria for defining AKI aligned with the Kidney Disease Improving Global Outcomes (KDIGO) criteria in nine publications (18–26). In the remaining three studies (27–29), specific definitions of AKI were not explicitly outlined. Recovery from AKI was defined as a serum creatinine value within 20% (30) of the baseline level obtained within 90 days after peak serum creatinine (19), or by hospital discharge (23). The term “ventilator therapy” was defined as invasive mechanical ventilation. To assess the risk of bias risk in the included studies, we utilized the Newcastle-Ottawa Scale (NOS) (31). This scale evaluates studies based on three main aspects: selection (0–4 stars), comparability (0–2 stars), and exposure between the case and control groups (0–3 stars). Scores of 3 or lower indicate poor quality, scores of 4 to 6 denote moderate quality, and scores of 7 to 9 signify high quality. The strength of evidence regarding the primary and secondary outcomes was graded according to the Grading of Recommendations, Assessment, and Evaluation (GRADE) system (32). The certainty of evidence for each outcome was categorized as high, moderate, low, or very low.

### 2.4. Subgroup analysis

Subgroup analyses related to AKI were conducted considering factors such as the types of influenza (type A vs. type A or B), age (< 65 vs. ≥ 65 years), distribution of gender (female <50% vs. ≥ 50%), ICU admission rate (< 100% vs. 100%), occurrence of shock (< 50% vs. ≥ 50%), and utilization of vasopressors (< 50% vs. ≥ 50%).

### 2.5. Data synthesis and statistical analysis

Patients with COVID-19 infection were compared to those with influenza infection. The effect size was quantified using the pooled odds ratio (OR) along with a 95% confidence interval (CI). Some studies (18, 20, 21, 24, 25, 27–29) reported the length of hospital or ICU stays as a median with a range. To consolidate data, we estimated the mean and variance using a validated formula based on the reported median, range, and sample size (33). The possibility of publication bias was assessed through funnel plots and Egger’s test. Heterogeneity between trials was evaluated using the I^2^ test, where values exceeding 50% were indicative of substantial heterogeneity. To explore reasons for differences among COVID-19/influenza patients or across studies, a meta-regression was performed. Comprehensive Meta-Analysis (Version 3.3.070, 20 November 2014) was employed for all statistical analyses. Statistical significance was defined as *p*-values below 0.05.

## 3. Results

### 3.1. Literature search and included patients

**Figure 1:**
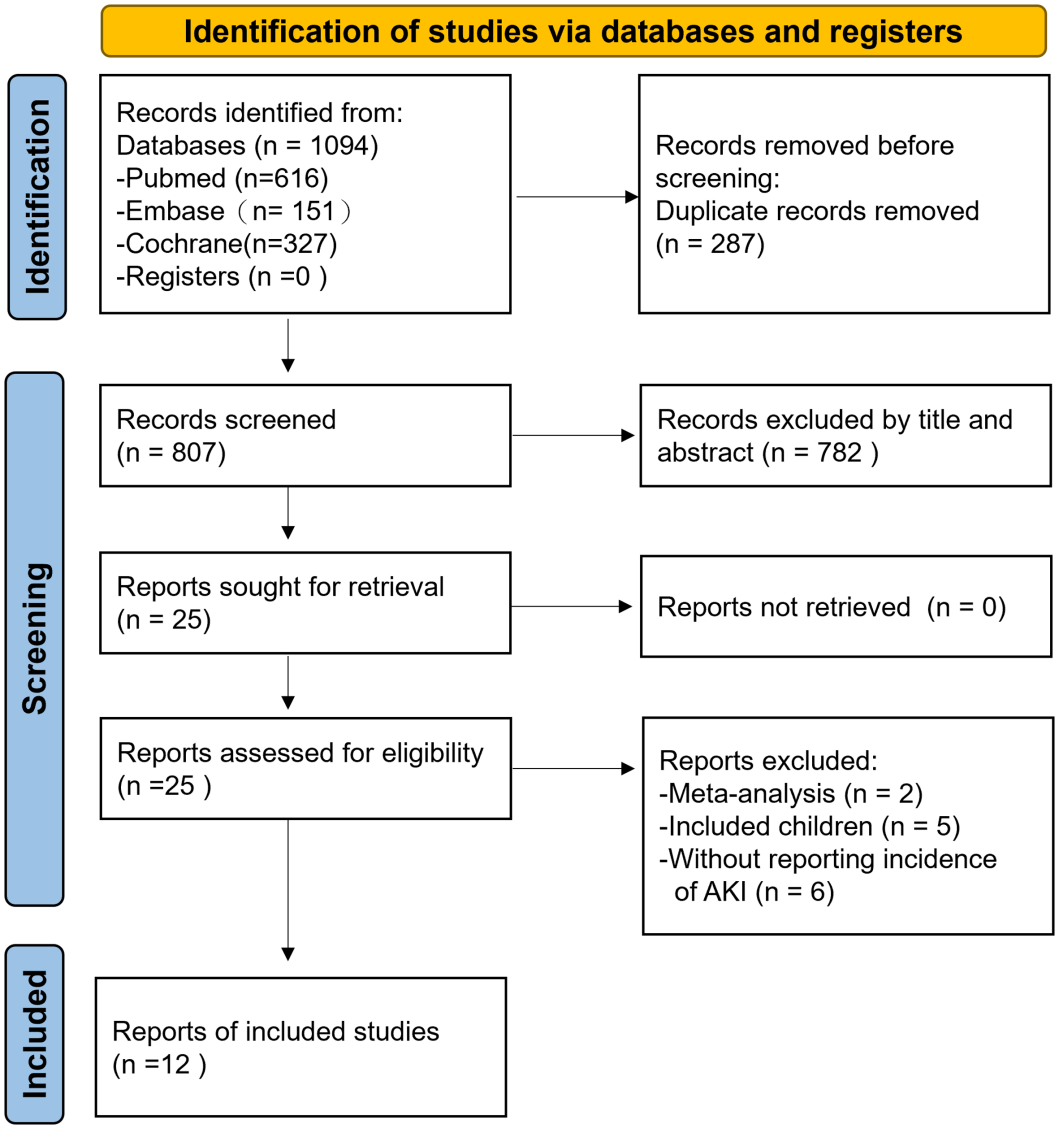
Flow diagrams of the studies selection. AKI, acute kidney injury.

As shown in Figure 1, 1,094 studies were identified through the database search. After removing 287 duplicate articles, 807 articles were screened with titles and abstracts, and 782 records were excluded as they did not meet the inclusion criteria (“PICOS”) in our work. We assessed 25 full-text articles for eligibility, of which 13 were excluded: one was a meta-analysis involving no influenza patients, one was a meta-analysis involving other viral infections in addition to influenza (8), five included only children or patients under 18 years of age (34–38), and six did not report the incidence of AKI. Ultimately, 12 studies (18–29) with total 17,618 participants were included in the meta-analysis. The search equation is listed in Supplement Text 1, and the characteristics of the included studies are provided in Table 1.

**Table 1:** Summary of the baseline characteristics of the included studies.

Author	Nation	Patient population	Patient number (*n*)	Influenza type	*Age (years)	DM (%)	HTN (%)	CKD (%)	Shock (%)	Female (%)	ICU admission COVID-19/influenza (%)
Bhasin et al. (18)	United States	Hospitalization	758	NR	68.6 (56–81)^a^	35.62	54.61	22.03	11.47	55.27	43.4/38.1
Birkelo et al. (19)	United States	Hospitalization	7,082	NR	67.3 (13.5)	51	84	NR	6.96	6.6	NR
Cobb et al. (21)	United States	ICU	139	A or B	58.5 (16.7)	33.81	NR	16.54	51.79	36.69	100/100
Cousin et al. (20)	France	ICU with ARDS on ECMO	52	A or B	56.2 (47–61)^a^	23.07	44.23	NR	59.61	26.92	100/100
Erich et al. (22)	Vienna, Austria	Hospitalization	708	A or B	73.5 (61–82)^a^	24.9	NR	29.94	NR	49.71	8.5/6.5
Lachant et al. (24)	United States	Hospitalization	924	A or B	64.7 (48–75)^a^	35.82	65.47	23.16	10.49	53.24	34.5/13.5
Nasir et al. (28)	Pakistan	Hospitalization	170	H1N1 (A)	55.6 (41–65)^a^	39.41	38.23	NR	17.05	35.29	14.8/27.3
Strohbehn et al. (23)	United States	Hospitalization	2,425	A or B	69.2 (17)	47.46	81.44	35.21	NR	53.03	NR
Tang et al. (27)	China	ICU with ARDS	148	H1N1 (A)	59.4 (49–68)^a^	23.64	47.29	6.08	22.28	29.05	100/100
Zheng et al. (29)	China	Hospitalization	505	H1N1 (A)	48.4 (32–63)^a^	7.46	17.05	4.69	16.63	42.85	5.2/4.1
Yildirim et al. (25)	Turkey	ICU	109	NR	64 (55–76)^a^	29.35	46.79	5.50	50.46	42.20	100/100
Shusterman et al. (26)	Israel	Hospitalization	4,634	A or B	71 (26.75)^a^	19.18	31.25	6.30	NR	46.98	NR

*Data of age are presented as mean ± standard deviation or ^a^: Median (interquartile range). ARDS, adult respiratory distress syndrome; CKD, chronic kidney disease; COVID-19, Coronavirus Disease 2019; DM, Diabetes Mellitus; ECMO, extracorporeal membrane oxygenation; HTN, hypertension; ICU, intensive care unit; NR, not reported.

### 3.2. Heterogeneity and quality of the enrolled studies

The 12 enrolled studies were published between 2020 and 2023. There were differences in the study populations (e.g., all critically ill patients in ICUs, ARDS requiring veno-venous ECMO support), sample size, observation period, and treatment strategies. The NOS score of the included studies was 8–9, except for one study with a score of 7 (27), which extracted data of the control population (influenza group) from a different institution. The quality of the included studies was considered high based on the NOS score (Supplement Table 1).

### 3.3. Characteristics of the included patients

A detailed overview of the study characteristics is presented in Table 1. All studies were retrospective cohort studies. Five studies were conducted in the United States (18, 19, 21, 23, 24), two in China (27, 29), one in France (20), one in Austria (22), one in Turkey (25), one in Israel (26), and one in Pakistan (28). Eight studies (18, 19, 22–24, 26, 28, 29) reported data on general hospitalized patients, and four studies reported data on critically ill patients in ICUs (20, 21, 25, 27) and patients with ARDS requiring veno-venous ECMO (20). Three studies (27–29) enrolled patients with H1N1 (subtype of influenza A), six studies (20–24, 26) enrolled patients with type A or B influenza, while the other four studies (18, 19, 24, 25) recruited influenza patients without mentioning the subtype. The meta-analysis included 8,634 patients with COVID-19 and 8,984 patients with influenza. The reported mean or median age ranged from 48.4 to 73.5 years. Overall, 31.83% of the total population were females (*n* = 5,609). The mean prevalence of pre-existing diabetes mellitus was 37.61% (*n* = 6,626) (range 7.46%–51.00%); the prevalence of hypertension ranged from 17.05% to 84.00%, and the prevalence of CKD varied from 4.69% to 35.21%. Detailed outcomes of the included studies are presented in Table 2.

**Table 2:** Summary of the outcomes for the included studies.

Author	AKI COVID-19/influenza (%)	Recovery from AKI COVID-19/influenza (%)	In hospital mortality with AKI COVID-19/influenza (%)	ARDS COVID-19/influenza (%)	Ventilator therapy COVID-19/ influenza (%)	ECMO COVID-19/influenza (%)	Length of hospital stay COVID19/influenza (days)^#^	Length of ICU stay COVID-19/influenza (days)^#^
Bhasin et al. (18)	32.6/33	NR	11.3/3.5	NR	12.9/6.5	NR	6 (3–12)/4 (2–6)	NR
Birkelo et al. (19)	44.6/27.7	57.3/82.3	32.1/3.9	NR	16.7/3.1	NR	14.7 (20.0)*/5.4 (10.1)*	NR
Cobb et al. (21)	43.1/40.5	NR	NR	63/25.7	61.5/94.6	NR	14 (8–22)/8 (5–23)	9 (3–16)/4 (2–14)
Cousin et al. (20)	50/54.5	NR	NR	NR	100/100	100/100	29 (21–47)/33 (23–45)	27 (20–39)/31 (22–38)
Erich et al. (22)	27.5/12.4	NR	NR	NR	NR	NR	NR	NR
Lachant et al. (24)	43.4/29.4	NR	NR	NR	19.4/4.9	NR	8 (3–21)/4 (2–7)	8 (3–18)/6 (3–11)
Nasir et al. (28)	20.9/40	NR	NR	24.5/30.9	41.7/69.1	NR	7 (4–10)/6 (4–10)	NR
Strohbehn et al. (23)	23/13.4	55.8/70.4	32.3/10.6	NR	NR	NR	9.9 (10.8)*/5.7 (5.7)*	NR
Tang et al. (27)	17.8/10.7	NR	NR	100/100	19.2/85.5	13.7/33.3	13 (10–18)/16 (9–30)	NR
Zheng et al. (29)	2.8/2.3	NR	NR	4.4/2.3	4.4/2.7	NR	17.5 (13–24)/7 (4–10)	NR
Yildirim et al. (25)	29.6/65.5	NR	NR	NR	42.6/65.5	NR	18 (11–29)/24 (13–42)	12 (5–18)/12 (6–29)
Shusterman et al. (26)	11.8/13.3	NR	NR	NR	12.4/6.5	NR	NR	NR

^#^ Data of length of hospital stay are presented as median, interquartile range. *Data of length of hospital stay are presented as mean ± standard deviation. AKI, acute kidney injury; ARDS, adult respiratory distress syndrome; COVID-19, Coronavirus disease 2019; ECMO, extracorporeal membrane oxygenation; ICU, intensive care unit; NR, not reported.

### 3.4. Primary outcome: the incidence of AKI

**Figure 2:**
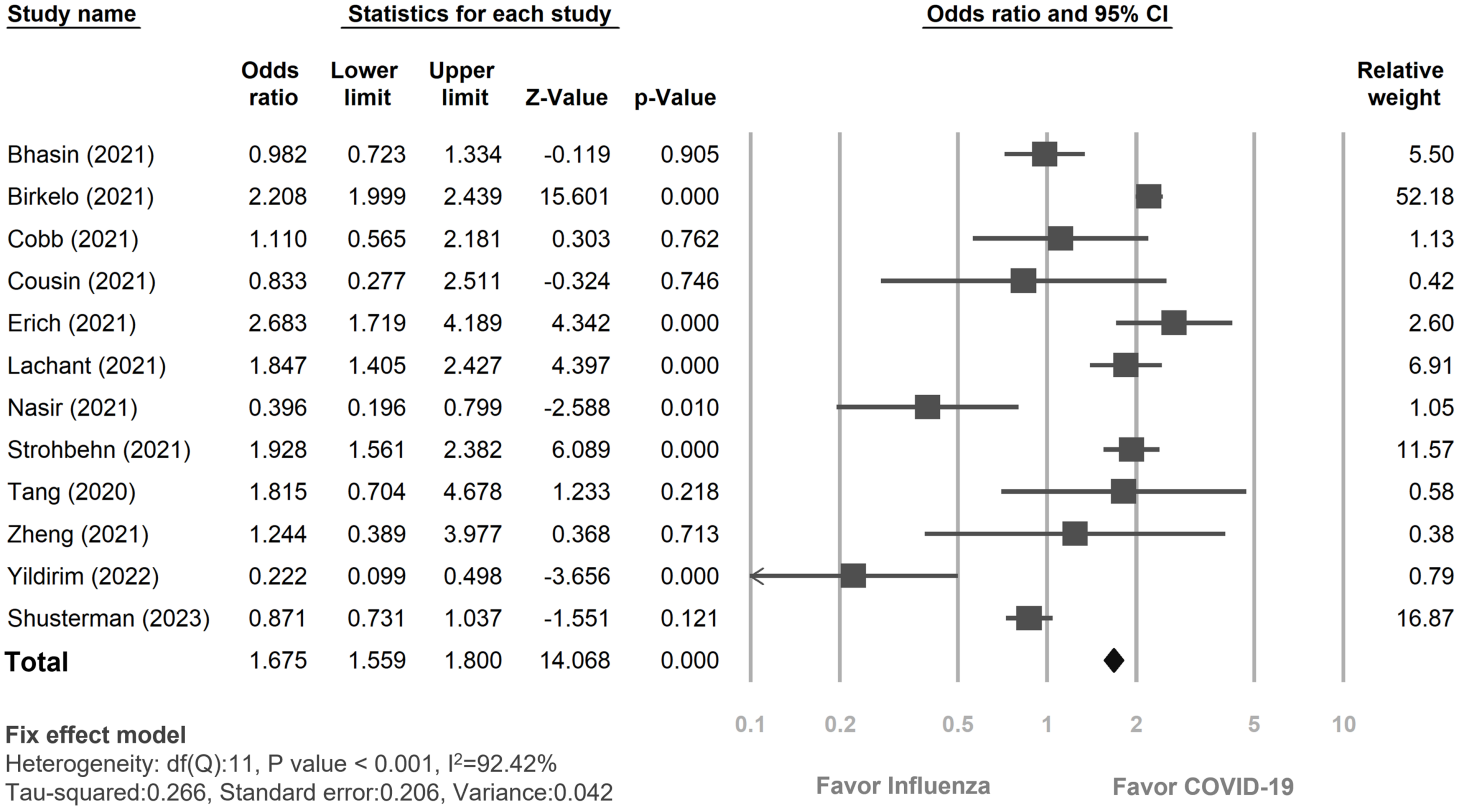
Forest plot depicted the risk of incidence of acute kidney injury between COVID-19 and influenza patients. CI, confidence interval; COVID-19, coronavirus disease 2019.

The pooled incidence of AKI was significantly higher in COVID-19 infection (2,536 out of 8,634, 29.37%) compared to those with influenza infection (1,855 out of 8,984, 20.98%). This yielded an odds ratio (OR) of 1.67 (95% CI 1.56–1.80, *p* < 0.01; Figure 2). However, significant degree of heterogeneity was observed (I^2^ = 92.42%). In subgroup analyses (Supplement Figure 1), the incidence of AKI development was notably greater in the COVID-19 group than in the influenza group, including both type A and B (OR: 1.27, 95% CI 1.12–1.44, *p* < 0.01). Yet, the difference was insignificant when comparing to those with solely influenza A (OR: 0.76, 95% CI 0.46–1.67, *p* = 0.29) (27–29). Regarding the impact of age on the incidence of AKI, among the studies which recruited relatively older patients (i.e., age ≥ 65 years) (18, 19, 22, 23, 26), COVID-19 infection was associated with a significantly higher risk of AKI compared to influenza infection (OR: 1.74, 95% CI 1.61–1.88, *p* < 0.01). Similarly, studies involving relatively younger patients (i.e., age < 65 years) also demonstrated a trend towards a heightened risk of AKI incidence in COVID-19 patients relative to those with influenza infection (OR: 1.25, 95% CI 1.01–1.55, *p* = 0.04). Gender did not impact the observed higher risk of AKI incidence in the COVID-19 group when compared to the Influenza group (Female <50%, OR: 1.69, 95% CI 1.56–1.84, *p* < 0.01; Female ≥50%, OR: 1.63, 95% CI 1.48–1.89, *p* < 0.01). However, among studies involving relatively critical patients, there was no significant difference in the incidence of AKI between COVID-19 infection and Influenza infection (i.e., ICU admission = 100%, OR: 0.76, 95% CI 0.50–1.15, *p* = 0.20; shock ≥50%, OR: 0.61, 95% CI 0.38–0.98, *p* = 0.04; vasopressors therapy ≥50%, OR: 1.03, 95% CI 0.58–1.83, *p* = 0.93).

### 3.5. Secondary outcomes

#### 3.5.1. In-hospital mortality with AKI

**Figure 3:**
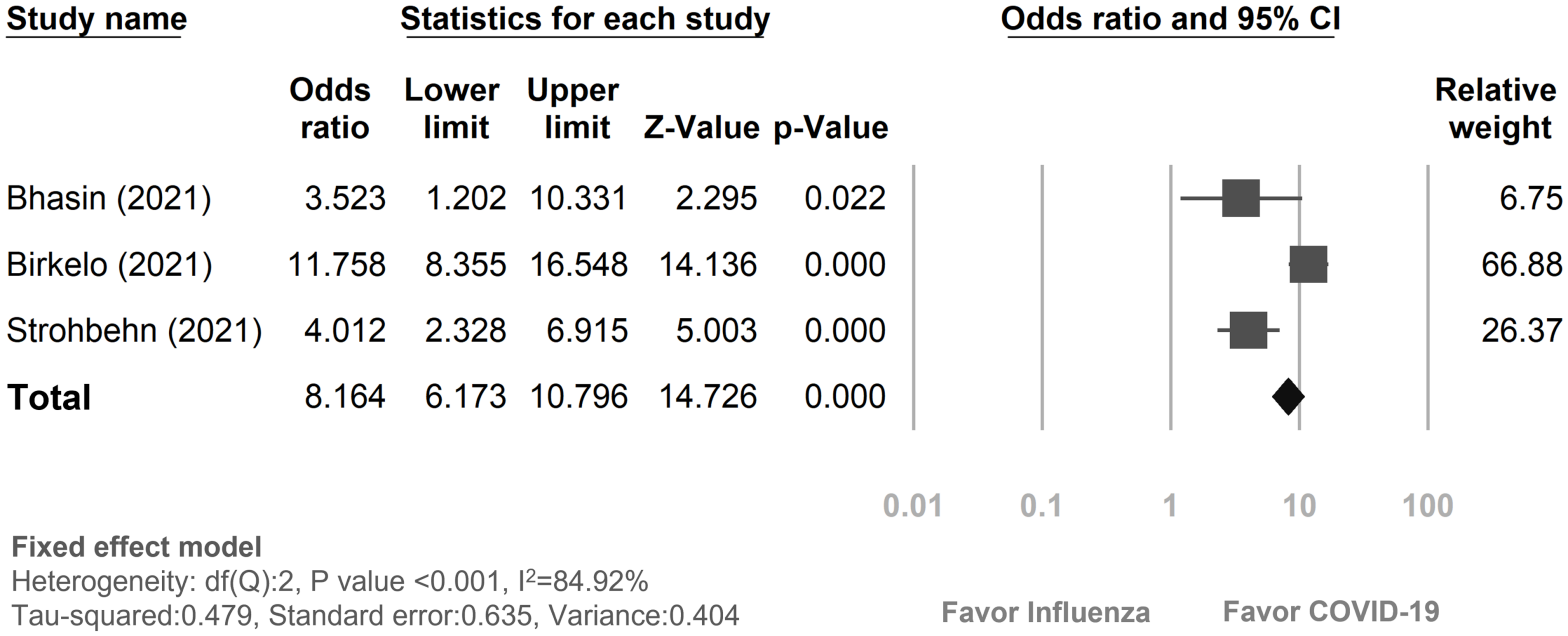
Forest plot depicted the risk of in-hospital mortality with acute kidney injury between COVID-19 and influenza patients. CI, confidence interval; COVID-19, coronavirus disease 2019.

Three studies (18, 19, 23) provided data on in-hospital mortality rates among COVID-19 and influenza patients with AKI (Figure 3). The mortality rate in COVID-19 patients with AKI during hospitalization was 30.95% (580 out of 1,874), while it was 5.51% (62 out of 1,126) in influenza patients. The risk of in-hospital mortality was pronouncedly higher in COVID-19 patients with AKI than the influenza patients with AKI (OR: 8.16, 95% CI 6.17–10.80, *p* < 0.01, I^2^ = 84.92%, Figure 3).

#### 3.5.2. Recovery from AKI

**Figure 4:**
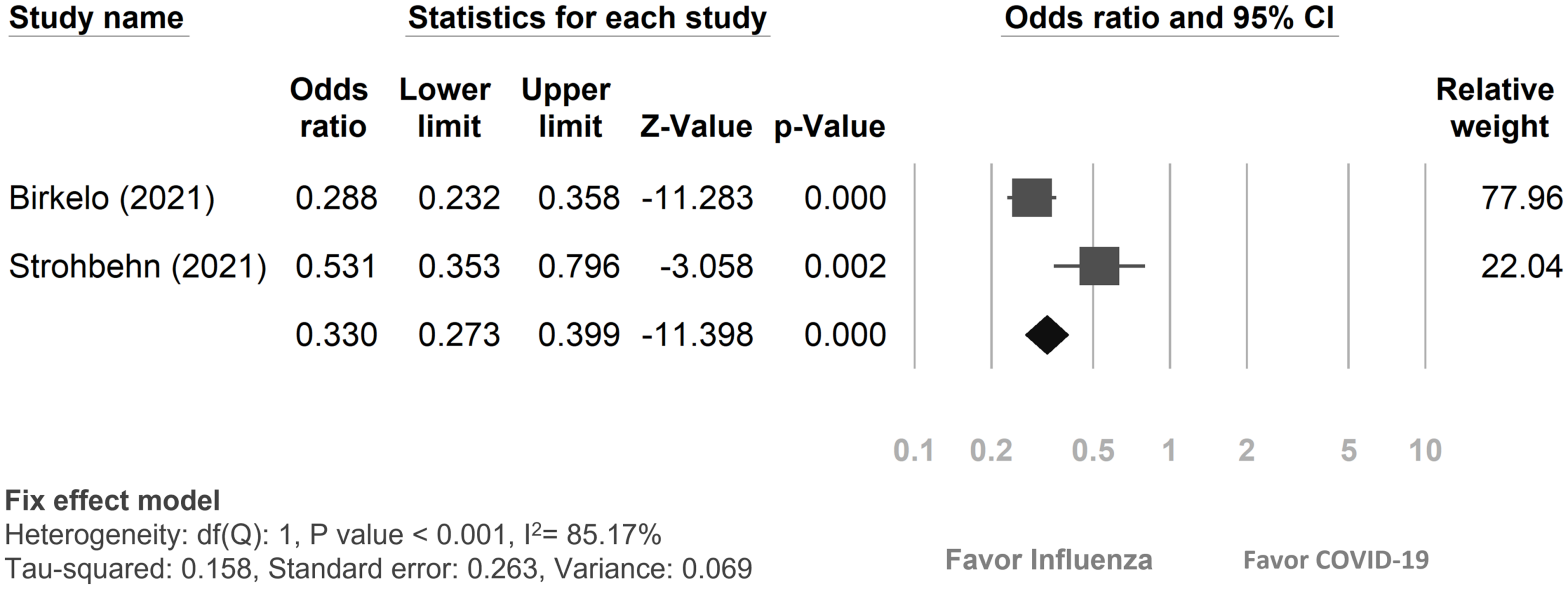
Forest plot depicted the outcome of recovery from acute kidney injury between COVID-19 and influenza patients. CI, confidence interval; COVID-19, coronavirus disease 2019.

Among the 12 studies included, only two (19, 23) reported on the recovery rate from AKI (Figure 4). The findings revealed that 57.02% (727 out of 1,275) of COVID-19 patients experienced a recovery from AKI, while 80.23% (820 out of 1,022) of influenza patients with AKI returned to their baseline kidney function. In comparison, patients with COVID-19 patients were less likely to recover from AKI compared to those with influenza (OR: 0.33, 95% CI 0.27–0.40, *p* < 0.01, I^2^ = 85.17%).

#### 3.5.3. Length of hospital/ICU stay

**Figure 5:**
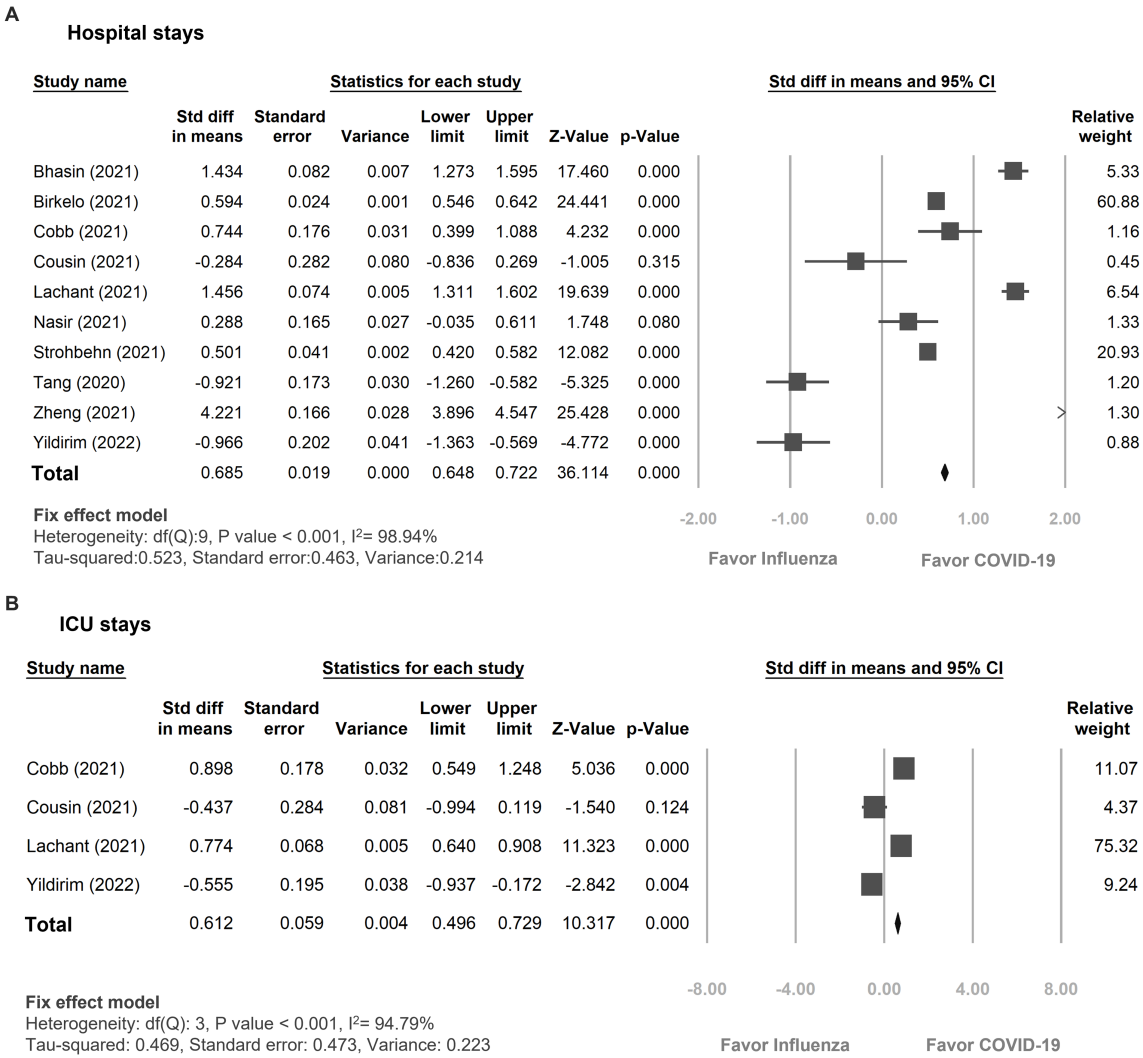
Forest plot showing the outcomes of **(A)** hospital days **(B)** ICU days between the patients with COVID-19 and influenza infection. CI, confidence interval; COVID-19, coronavirus disease 2019.

The duration of hospitalization was remarkably extended in the COVID-19 group compared to the influenza group, displaying a standardized mean difference of 0.69 days (95% CI 0.65–0.72, *p* < 0.01, I^2^ = 98.94%, Figure 5A). Additionally, patients with COVID-19 infection experienced a longer stay in the ICU compared to patients with influenza, with a standardized mean difference of 0.61 days (95% CI 0.50–0.73, *p* < 0.01, I^2^ = 94.80%, Figure 5B).

#### 3.5.4. Renal replacement therapy

The incidence of RRT was reported in five studies (19, 21, 24–26). When these findings were pooled, the COVID-19 group exhibited an incidence of 4.10% (271 out of 6,609), while the influenza group showed an incidence of 1.51% (95 of 6,279). COVID-19 patients were at a significantly higher risk of requiring RRT in comparison to those with influenza (OR: 2.17, 95% CI 1.66–2.85, *p* < 0.01, I^2^ = 94.87%, Supplement Figure 2).

#### 3.5.5. Vasopressor therapy, ventilator therapy and ARDS

Overall, patients with COVID-19 infection were found to have a heightened risk of vasopressor therapy (14.44% vs. 4.32%, OR: 3.40, 95% CI 2.83–4.08, *p* < 0.01, I^2^ = 94.82%, Supplement Figure 3), ventilator therapy (16.14% vs. 7.43%, OR: 2.62, 95% CI 2.30–2.97, *p* < 0.01, I^2^ = 96.51%, Supplement Figure 4), as well as the development of ARDS (30.28% vs. 27.36%, OR: 1.87, 95% CI 1.18–2.94, *p* < 0.01, I^2^ = 79.36%, Supplement Figure 5) in comparison to patients with influenza.

### 3.6. Heterogeneity and publication bias

The I-Square statistic indicated substantial heterogeneity among the 12 included studies for the incidence of AKI, with a value of 92.42%. Subgroup analysis was conducted to explore potential sources of heterogeneity, and consistent patterns were observed across different age groups (<65 years old or ≥ 65 years old) and gender distributions (i.e., female <50% or ≥ 50%). However, variations in disease severity factors, such as the proportion of ICU admission (i.e., <100% or 100%), shock occurrence (i.e., <50% or ≥ 50%), and the usage of vasopressors (i.e., <50% or ≥ 50%), yielded differing outcomes between the COVID-19 and influenza groups. While the Egger’s test did not indicate statistically significant evidence of publication bias (intercept: −2.56, standard error: 1.49, 95% CI: −5.87 to 0.76, *t*-value: 1.72, *p*-value: 0.12), the funnel plot displayed an asymmetric distribution (see Supplement Figure 6). This discrepancy suggests that although the Egger’s test did not find significant publication bias, the visual inspection of the funnel plot raises concerns about its potential presence regarding AKI. It is worth noting that Egger’s test has limitations and might not always detect subtle or complex forms of bias. Therefore, the possibility of publication bias should not be entirely dismissed and warrants further investigation.

### 3.7. Meta-regression analysis

The meta-regression bubble plot demonstrated that hypertension was linked to a higher AKI risk between the COVID-19 and influenza groups (*Z* = 3.79; *p* < 0.01, Supplement Figure 7C). However, no significant differences were observed in relation to age (*Z* = 1.39; *p* = 0.16, Supplement Figure 7A), pre-existing diabetes mellitus (*Z* = 0.39; *p* = 0.23, Supplement Figure 7B), chronic kidney disease (*Z* = 2.23; *p* = 0.03, Supplement Figure 7D), coronary artery disease (*Z* = 0.60; *p* = 0.55), gender (*Z* = −0.37; *p = 0.71*), rate of ICU admission (*Z* = 0.21; *p = 0.84*), or the presence of shock (*Z* = 0.26; *p = 0.80*).

### 3.8. Quality and GRADE assessment

We examine the quality of evidence using NOS quality assessment, which showed a score for the included studies of 7–9 (Supplement Table 1). GRADE assessment revealed moderate certainty for in-hospital mortality, and low certainty for the incidence of AKI and recovery from AKI (Supplement Table 2).

## 4. Discussion

Our analysis of 12 cohort studies indicated that patients with COVID-19 were at a heightened risk of developing AKI compared to patients with influenza, irrespective of age or gender. However, no significant difference between the two groups was observed in subgroup analyses among critically ill patients, such as those who presented with shock, required vasopressor therapy, or had ICU admission. The difference of AKI incidence was also unremarkable when comparing to those with solely influenza A. In addition, the patients with COVID-19 had a noticeably higher risk of in-hospital mortality with AKI, lower recovery rate from AKI to baseline kidney function, longer hospital stay/ICU stay, requiring RRT, vasopressor utilization, ventilator support and occurrence of ARDS compared to the patients with influenza. Our findings diverged from a previous meta-analysis conducted by Cau (8), which primarily focused on critically ill patients. Their study compared the frequencies of AKI and RRT among individuals with COVID-19, SARS, Middle East Respiratory Syndrome (MERS), and influenza. Cau categorized the data into three groups: COVID-19, ACE-2 associated viral infection (SARS, influenza H1N1, influenza H7N9), and non-ACE-2 associated viral infection (MERS, influenzas other than H1N1, H7N9). In critically ill patients, AKI frequencies did not display significant differences across virus groups: COVID-19 (51%), ACE2-associated respiratory viruses (56%), and non-ACE2-associated viruses (63%), nor between ACE2-associated respiratory viruses and non-ACE2-associated viruses (*p* = 0.624). This could potentially be attributed to selection bias. Shock, including septic shock arising from bacterial sepsis, and the use of vasopressors are common antecedents and contributors to AKI in critically ill patients. This is due to the changes in total renal blood flow and the distribution of renal blood flow caused by shock and the utilization of vasopressor. Additionally, these conditions result in elevated levels of pro-inflammatory cytokines and other mediators associated with AKI (39, 40).

In our study, the higher incidence rate of AKI in the patients with COVID-19 than in those with influenza (29.37% vs. 20.98%, OR: 1.67) may be attributed to viral tropism of SARS-CoV-2 (41) in the kidney by the molecular pathways associated with angiotensin converting enzyme 2 (ACE2). The SARS-CoV-2 enters the host cells through the enzymatic receptor of ACE2 and transmembrane protease, serine 2. These receptors are highly expressed not only in lung, but also in renal proximal tubules and podocytes, as well as other organs such as the heart, intestine, stomach, and esophagus (42, 43). In addition, the pathogenesis in COVID-19 patients with AKI involves both the direct and indirect effects of the SARS-CoV-2 virus on the kidney (5, 28). Direct cytopathic effects of a replicating virus, inflammation-induced cell injury from infiltrating inflammatory cells, acute tubule necrosis and podocyte damage contribute to protein leakage from the Bowman’s capsule and occurrence of collapsing glomerulopathy (43). COVID-19 infection can activate complement (C5b-9) deposition at the brush borders of apical tubules, leading to cell apoptosis, tubulointerstitial damage and impairment of renal function (44, 45). Cytotoxic particles such as perforin, granulysin released by activated killer lymphocytes and leukocyte-derived proinflammatory cytokines such as interleukin-1, interleukin-6 and tumor necrosis factor-alpha, further lead to a cytokine storm. The cytokine storm induces cellular apoptosis and pyroptosis, thus accelerating tubular damage and interstitial fibrosis. Moreover, increased secretion of interleukin-6 activates vascular endothelial growth factor, reduces the expression of E-cadherin, and enhances vascular permeability, resulting in capillary leak syndrome (46). Furthermore, COVID-19 infection can activate coagulation factors resulting in microvascular thrombosis, and macrophages, which can lead to erythro-phagocytosis and anemia (5, 45, 47). Additionally, COVID-19 infection can impair the renin–angiotensin–aldosterone system (RAAS). The binding of the SARS-CoV-2 virus to its ACE2 receptor contributes to the loss of ACE2 catalytic activity in the RAAS, leading to the upregulation of angiotensin II, which exerts pro-inflammatory, pro-fibrotic, and anti-diuretic and vasoconstrictive properties by activating the Ang T1 receptor (43, 46, 48). Collectively, these mechanisms contribute to AKI through a direct viral effect on the kidneys.

The indirect effects of COVID-19 infection, including systemic consequences of viral infection (e.g., sepsis, volume depletion, hypoxia, rhabdomyolysis, and secondary infection with other viruses, bacteria, and fungi), nephrotoxicity associated with drugs (e.g., antibiotics) and organ crosstalk can also contribute to AKI (5, 43). Organ crosstalk refers to the intricate biological communication between distant organs by the release of signaling factors like cytokines and growth factors, as well as the release of damage-associated molecular patterns from injured tissues. SARS-CoV-2 infection may potentially induce ARDS, and inadequate of lung function may lead to hypoxia of the renal medulla and hypercapnic acidosis. These conditions can contribute to acute tubular necrosis and renal failure by diminishing the glomerular filtration rate. Dysfunction of the cardiovascular system, manifested as acute myocarditis, myocardial infarction, and heart failure due to SARS-CoV-2 infection, can lead to a reduction in cardiac output, leading to a decrease in glomerular filtration rate (5, 49).

Unlike SARS-CoV-2, which has been demonstrated to exhibit renal tropism, the influenza virus possesses viral-specific tropism for respiratory epithelial cells. It enters host cells by binding to α 2,6-linked sialic acid receptors through hemagglutinin, a surface glycoprotein expressed on the viral envelope (50, 51). Acute tubular necrosis has been identified as the primary histological finding in renal biopsies of patients infected with influenza A (52, 53). This condition arise from various factors, including sepsis-related hypovolemia, drug toxicity, aggressive fluid accumulation (54), and potentially the occurrence of rhabdomyolysis (55). Glomerulonephritis and thrombotic microangiopathy have also been proposed, yet neither of these conditions have evidence of influenza presence in kidney epithelial and/or glomerular cells (56). Renal tropism and direct viral effects on the kidneys may partially elucidate why the incidence of AKI is higher in COVID-19 patients compared to those with influenza infection.

However, the incidence of AKI between the COVID-19 patients and those with influenza A infection (but not those with influenza B infection) was not significantly different in subgroup analysis in this study. This may be because influenza type A viruses are known to cause pandemics and usually cause severe disease mostly in older people and those with complicated underlying disease, whereas patients with influenza B usually have less severe symptoms. Evidence of renal tropism has also been observed in cohorts of influenza H1N1 patients (52). In addition, with regards to genetic and antigenic characterization, influenza A viruses are known to undergo a process called “antigenic shift,” which has resulted in the emergence of novel influenza A viruses with a hemagglutinin that is genetically and antigenically distinct from previously circulating strains (4, 57). Seasonal influenza A and B viruses primarily evolve to escape human humoral immunity via antigenic drift, a process of genetic change or mutation that involves with amino acid substitutions, insertions, or deletions coding for hemagglutinin and neuraminidase epitopes. Influenza B typically undergoes less rapid genetic changes than influenza A, resulting in less antigenic drift. As a result, many adults may have significant immunity to influenza B, and there is usually no significant difference in the incidence of AKI between patients with COVID-19 and those with influenza A infection (58, 59).

In this study, the patients with COVID-19 and AKI had a significantly higher rate of in-hospital mortality compared to those with influenza. This difference may be due to the increased knowledge and implementation of preventative and treatment strategies for influenza, such as vaccination and antiviral medications, which can reduce the severity of the disease and decrease the likelihood of complications such as AKI. Regarding the preventive strategies, the influenza vaccination coverage rate for adults aged 18 years and older was between 41.7% ~ 50.2% during influenza seasons between 2019 and 2021 in the United States (60), while the first COVID-19 vaccine was developed and authorized in late 2020. Regarding treatment, there are effective antiviral agents and well established guidelines for the antiviral treatment of influenza published by the US Centers for Disease Control and Prevention (61) and the Infectious Diseases Society of America (62). Three classes of antiviral drugs are available for influenza: (1) neuraminidase inhibitors (oseltamivir, zanamivir, and peramivir); (2) cap-dependent endonuclease inhibitors (baloxavir); and (3) adamantanes (amantadine and rimantadine). Most of the evidence suggests that the use of antiviral therapy, such as oseltamivir, can shorten the duration of influenza symptoms, reduce the duration of viral shedding, relieve illness severity, and decrease rates of complication, hospital admissions, and length of hospital stays (63). For hospitalized patients with suspected or confirmed influenza, the initiation of antiviral treatment is recommended as soon as possible; however, studies on antiviral agents for COVID-19 are still ongoing.

Another important finding of this study is that fewer of the patients with COVID-19 recovered from AKI compared to those with influenza This difference may be due to the renal tropism of SARS-COV-2 and a more severe pathophysiological course of AKI as mentioned above. Additionally, the greater number of comorbidities, more advanced stages of AKI, higher severity of the overall disease, and higher mortality rates in the COVID-19 group (18, 19) along with the lack of effective treatment for COVID-19, may also have contributed to the poor recovery from AKI.

## 5. Strengths and limitations

This study is the first comprehensive meta-analysis to investigate differences in the incidence of AKI, the recovery rate from AKI, the in-hospital mortality associated with AKI, and the length of hospital stay between patients with COVID-19 and those with influenza infection. These findings shed light on the importance of early interventions for renal protection in COVID-19 infected patients. This involves implementing public health policies for preventive vaccinations, taking steps to minimize additional renal toxicity insults, ensuring appropriate fluid management to prevent dehydration caused by gastrointestinal symptoms, and rigorously monitoring renal function throughout the duration of hospitalization. However, this study has several limitations. First, most studies enrolled COVID-19 and influenza patients at different periods attributable to the seasonal epidemics of influenza. The predominant influenza strain differed annually, and the pathogenicity of the different strains also varied in terms of disease severity. Second, some studies failed to provide a specific definition of AKI, and the criteria for baseline serum creatinine levels differed across studies. The timing of AKI was either generally unspecified or spanned a wide time range, potentially leading to information bias. Third, significant heterogeneity was observed among the included studies, characterized by factors such as diverse follow-up periods and varied degrees of disease severity. The asymmetric funnel plot suggest a lack of small-scale studies investigating the risk of AKI in COVID-19 patients; therefore, additional small-scale research is warranted to further assess the risk of AKI in patients with COVID-19. However, we report several important findings from our subgroup analyses enhancing their applicability across different contexts. Future studies are expected to offer further clarity regarding the definition and specific timing of AKI development. Additionally, an important limitation to consider is the dramatic shift in attention towards COVID-19 since late 2019, which has resulted in a higher number of publications and screenings for COVID-19 compared to influenza. This could introduce a selection bias into our meta-analysis. Additionally, the widespread implementation of COVID-19 preventive measures may have incidentally reduced the incidence of influenza, affecting the comparability of the two diseases. It should be noted that the focus on COVID-19 has also led to increased testing and reporting, potentially inflating the incidence rates of associated conditions such as AKI.

Moreover, the SARS-CoV-2 strain is continually evolving, which results in corresponding changes in COVID-19 vaccination and treatment strategies. To address this, it becomes crucial to match either align the severity levels of infections or utilize propensity score matching when comparing results across various studies.

## 6. Conclusion

AKI was more commonly observed in patients hospitalized with COVID-19 than in those with influenza. This meta-analysis specifically highlights the lower recovery rate from AKI in the COVID-19 group. Additionally, there was a significantly higher rate of in-hospital mortality and longer hospital stay in the COVID-19 patients compared to those with influenza.

## Data availability statement

The original contributions presented in the study are included in the article/Supplementary material, further inquiries can be directed to the corresponding authors.

## Author contributions

J-YC: conceptualization, supervision, methodology, formal analysis, and writing – review and editing. C-CY: conceptualization and supervision and writing – review and editing. C-YH: project administration, literature search and review, registered at PROSPERO, data collection, data curation, visualization, writing original draft, and writing – review and editing. H-CP: methodology and writing – review and editing. V-CW: methodology. C-CS: literature review formal analysis and data curation. T-HY, M-HC, and K-CT: visualization. H-YW and W-CK: data curation and validation. All authors contributed to the article and approved the submitted version.

## Conflict of interest

The authors declare that the research was conducted in the absence of any commercial or financial relationships that could be construed as a potential conflict of interest.

## Publisher’s note

All claims expressed in this article are solely those of the authors and do not necessarily represent those of their affiliated organizations, or those of the publisher, the editors and the reviewers. Any product that may be evaluated in this article, or claim that may be made by its manufacturer, is not guaranteed or endorsed by the publisher.

## Supplementary material

The Supplementary material for this article can be found online at: https://www.frontiersin.org/articles/10.3389/fmed.2023.1252990/full#supplementary-material
