# Supplementary material for: Acute kidney injury in patients with COVID-19 compared to those with influenza: a systematic review and meta-analysis
Source: Front Med (Lausanne). 2023 Sep 19;10:1252990. doi: 10.3389/fmed.2023.1252990 (PMC10547056; doi:10.3389/fmed.2023.1252990)
Supplement: Supplementary file 1 [file Data_Sheet_1.pdf]

**Acute kidney injury in comparison with COVID-19 versus Influenza: a systematic review and meta-analysis**

Authors: Chiu-Ying Hsiao, Heng-Chih Pan, Vin-Cent Wu, Ching-Chun Su, Tzu-Hsuan Yeh, Min-Hsiang Chuang, Kuan-Chieh Tu, Hsien-Yi Wang, Wei-Chih Kan, Chun-Chi Yang, Jui-Yi Chen

# Supplementary appendix

This supplementary appendix provides:
**Supplement Text 1.** Search equation via PubMed, EMBASE, Cochrane
**Supplement Checklist**. PRISMA checklist.
**Supplement Text 2.** PROSPERO protocol registration.
**Supplement Figure 1.** Forest plot depicted subgroup analysis of influenza type (influenza A, influenza A or B), age ( <65 years old or ≥65 years old), sex (female, <50%, ≥50%), the proportion of ICU admission (<100%, 100%), shock (<50%, ≥50%) and using vasopressor(%) for the risk of acute kidney injury
**Supplement Figure 2.** Forest plot depicted the risk of renal replacement therapy between COVID-19 and influenza patients
**Supplement Figure 3.** Forest plot depicted the risk of Vasopressor therapy between COVID-19 and influenza patients
**Supplement Figure 4.** Forest plot depicted the risk of ventilator therapy between COVID-19 and influenza patients
**Supplement Figure 5.** Forest plot depicted the risk of acute respiratory distress syndrome between COVID-19 and influenza patients
**Supplement Figure 6.** Funnel plot depicted the risk of acute kidney injury between COVID-19 and influenza patients
**Supplement Figure 7.** Meta-regression bubble plot showing the effect modification of age, DM, hypertension, CKD and the risk of acute kidney injury according to COVID-19 and influenza infection.
**Supplement Table 1**. Newcastle-Ottawa Scale Quality Assessment of included studies
**Supplement Table 2.** Quality assessment the GRADE results

## Supplement Text 1. Search equation via PubMed, EMBASE, MEDLINE, and Cochrane library

**Appendix.**
Search strategies for the different databases ran on Aug 12, 2023.

**PubMed**
Search: (("COVID-19"[Mesh] OR "SARS-CoV-2"[Mesh]) AND ("Influenza, Human"[Mesh] OR "Influenza A Virus, H1N1 Subtype"[Mesh] OR "Influenza A Virus, H3N2 Subtype"[Mesh] OR "Influenza B virus"[Mesh] OR "Influenza A virus"[Mesh])) AND (("Acute Kidney Injury"[Mesh]) OR ("Patient Outcome Assessment"[Mesh] OR "Outcome Assessment, Health Care"[Mesh] OR "Outcome and Process Assessment, Health Care"[Mesh] OR "Treatment Outcome"[Mesh] OR "Critical Care Outcomes"[Mesh] OR "mortality" [Subheading] OR "Evidence-Based Practice"[Mesh] OR "Evidence-Based Emergency Medicine"[Mesh] OR "Renal Insufficiency, Chronic"[Mesh] OR "Causality"[Mesh] OR "Epidemiologic Factors"[Mesh] OR "Comorbidity"[Mesh] OR "Cohort Studies"[Mesh] OR "Risk"[Mesh] OR "Prognosis"[Mesh] OR "Mortality"[Mesh] OR "Kidney Failure, Chronic"[Mesh] OR "Death"[Mesh] OR "Long Term Adverse Effects"[Mesh])) Filters: from 2019/12/1 - 2023/8/12
(("COVID-19"[MeSH Terms] OR "SARS-CoV-2"[MeSH Terms]) AND ("influenza, human"[MeSH Terms] OR "influenza a virus, h1n1 subtype"[MeSH Terms] OR "influenza a virus, h3n2 subtype"[MeSH Terms] OR "Influenza B virus"[MeSH Terms] OR "Influenza A virus"[MeSH Terms]) AND ("Acute Kidney Injury"[MeSH Terms] OR ("Patient Outcome Assessment"[MeSH Terms] OR "outcome assessment, health care"[MeSH Terms] OR "outcome and process assessment, health care"[MeSH Terms] OR "Treatment Outcome"[MeSH Terms] OR "Critical Care Outcomes"[MeSH Terms] OR "Mortality"[MeSH Subheading] OR "Evidence-Based Practice"[MeSH Terms] OR "Evidence-Based Emergency Medicine"[MeSH Terms] OR "renal insufficiency, chronic"[MeSH Terms] OR "Causality"[MeSH Terms] OR "Epidemiologic Factors"[MeSH Terms] OR "Comorbidity"[MeSH Terms] OR "Cohort Studies"[MeSH Terms] OR "Risk"[MeSH Terms] OR "Prognosis"[MeSH Terms] OR "Mortality"[MeSH Terms] OR "kidney failure, chronic"[MeSH Terms] OR "Death"[MeSH Terms] OR "Long Term Adverse Effects"[MeSH Terms]))) AND (2019/12/1:2023/8/12[pdat])

**Translations**
**severe acute respiratory syndrome coronavirus 2:** "sars-cov-2"[MeSH Terms] OR "sars-cov-2"[All Fields] OR "severe acute respiratory syndrome coronavirus 2"[All Fields]
**SARS-CoV-2:** "sars-cov-2"[MeSH Terms] OR "sars-cov-2"[All Fields] OR "sars cov 2"[All Fields]
**Coronavirus disease 2019:** "covid-19"[MeSH Terms] OR "covid-19"[All Fields] OR "coronavirus disease 2019"[All Fields]
**COVID 19:** ("COVID-19" OR "COVID-19"[MeSH Terms] OR "COVID-19 Vaccines" OR "COVID-19 Vaccines"[MeSH Terms] OR "COVID-19 serotherapy" OR "COVID-19 serotherapy"[Supplementary Concept] OR "COVID-19 Nucleic Acid Testing" OR "covid-19 nucleic acid testing"[MeSH Terms] OR "COVID-19 Serological Testing" OR "covid-19 serological testing"[MeSH Terms] OR "COVID-19 Testing" OR "covid-19 testing"[MeSH Terms] OR "SARS-CoV-2" OR "sars-cov-2"[MeSH Terms] OR "Severe Acute Respiratory Syndrome Coronavirus 2" OR "NCOV" OR "2019 NCOV" OR (("coronavirus"[MeSH Terms] OR "coronavirus" OR "COV") AND 2019/11/01[PDAT] : 2020/08/12[PDAT]))
**Influenza:** "influenza's"[All Fields] OR "influenza, human"[MeSH Terms] OR ("influenza"[All Fields] AND "human"[All Fields]) OR "human influenza"[All Fields] OR "influenza"[All Fields] OR "influenzas"[All Fields] OR "influenzae"[All Fields]
**Influenza A virus:** "influenza a virus"[MeSH Terms] OR "influenza a virus"[All Fields]
**Influenza B virus:** "influenza b virus"[MeSH Terms] OR "influenza b virus"[All Fields]
**Acute kidney injury:** "acute kidney injury"[MeSH Terms] OR ("acute"[All Fields] AND "kidney"[All Fields] AND "injury"[All Fields]) OR "acute kidney injury"[All Fields]

**Acute renal failure:** "acute kidney injury"[MeSH Terms] OR ("acute"[All Fields] AND "kidney"[All Fields] AND "injury"[All Fields]) OR "acute kidney injury"[All Fields] OR ("acute"[All Fields] AND "renal"[All Fields] AND "failure"[All Fields]) OR "acute renal failure"[All Fields]
**Acute renal insufficiency:** "acute kidney injury"[MeSH Terms] OR ("acute"[All Fields] AND "kidney"[All Fields] AND "injury"[All Fields]) OR "acute kidney injury"[All Fields] OR ("acute"[All Fields] AND "renal"[All Fields] AND "insufficiency"[All Fields]) OR "acute renal insufficiency"[All Fields]

**Embase**
#1 [severe acute respiratory syndrome coronavirus 2.mp. [mp=ti, ab, tx, ct, sh, hw, tn, ot, dm, mf, dv, kf, fx, dq, bt, nm, ox, px, rx, an, ui, sy]
#2 [SARS-CoV-2.mp. [mp=ti, ab, tx, ct, sh, hw, tn, ot, dm, mf, dv, kf, fx, dq, bt, nm, ox, px, rx, an, ui, sy]

#3 [Coronavirus disease 2019.mp. [mp=ti, ab, tx, ct, sh, hw, tn, ot, dm, mf, dv, kf, fx, dq, bt, nm, ox, px, rx, an, ui, sy]
#4 [COVID-19.mp. [mp=ti, ab, tx, ct, sh, hw, tn, ot, dm, mf, dv, kf, fx, dq, bt, nm, ox, px, rx, an, ui, sy]
#5 [Human Influenza.mp. [mp=ti, ab, tx, ct, sh, hw, tn, ot, dm, mf, dv, kf, fx, dq, bt, nm, ox, px, rx, an, ui, sy]
#6 [Influenza virus A.mp. [mp=ti, ab, tx, ct, sh, hw, tn, ot, dm, mf, dv, kf, fx, dq, bt, nm, ox, px, rx, an, ui, sy]
#7 [Influenza virus B.mp. [mp=ti, ab, tx, ct, sh, hw, tn, ot, dm, mf, dv, kf, fx, dq, bt, nm, ox, px, rx, an, ui, sy]
#8 [H1N1.mp. [mp=ti, ab, tx, ct, sh, hw, tn, ot, dm, mf, dv, kf, fx, dq, bt, nm, ox, px, rx, an, ui, sy]
#9 [H3N2.mp. [mp=ti, ab, tx, ct, sh, hw, tn, ot, dm, mf, dv, kf, fx, dq, bt, nm, ox, px, rx, an, ui, sy]
#10[Acute kidney injury.mp. [mp=ti, ab, tx, ct, sh, hw, tn, ot, dm, mf, dv, kf, fx, dq, bt, nm, ox, px, rx, an, ui, sy]
#11
[Acute renal failure.mp. [mp=ti, ab, tx, ct, sh, hw, tn, ot, dm, mf, dv, kf, fx, dq, bt, nm, ox, px, rx, an, ui, sy]
#12 [acute renal insufficiency.mp. [mp=ti, ab, tx, ct, sh, hw, tn, ot, dm, mf, dv, kf, fx, dq, bt, nm, ox, px, rx, an, ui, sy]
#13[renal outcome.mp. [mp=ti, ab, tx, ct, sh, hw, tn, ot, dm, mf, dv, kf, fx, dq, bt, nm, ox, px, rx, an, ui, sy]
#14 [outcomes.mp. [mp=ti, ab, tx, ct, sh, hw, tn, ot, dm, mf, dv, kf, fx, dq, bt, nm, ox, px, rx, an, ui, sy]
#15 [complications.mp. [mp=ti, ab, tx, ct, sh, hw, tn, ot, dm, mf, dv, kf, fx, dq, bt, nm, ox, px, rx, an, ui, sy]
#16[mortality.mp. [mp=ti, ab, tx, ct, sh, hw, tn, ot, dm, mf, dv, kf, fx, dq, bt, nm, ox, px, rx, an, ui, sy]
(#1 or #2)
#17[hospital mortality.mp. [mp=ti, ab, tx, ct, sh, hw, tn, ot, dm, mf, dv, kf, fx, dq, bt, nm, ox, px, rx, an, ui, sy]
#18 [1 or 2 or 3 or 4]
#19[5 or 6 or 7 or 8 or 9]
#20 [10 or 11 or 12 or 13 or 14 or 15 or 16 or 17]
#21 [limit 20 to full text]
#22 [limit 21 to [01-12-0219]/sd NOT [13-08-2023]/sd AND [2019-2023]/py

**Cochrane**
#1 MeSH descriptor: [COVID-19] explode all trees
#2 MeSH descriptor: [SARS-CoV-2] explode all trees
#3 MeSH descriptor: [Influenza] explode all trees
#4 MeSH descriptor: [H1N1] explode all trees
#5 MeSH descriptor: [H3N2] explode all trees
#6 MeSH descriptor: [Acute Kidney Injury] explode all trees
#7 MeSH descriptor: [Acute renal failure] explode all trees
#8 MeSH descriptor: [Acute renal insufficiency] explode all trees
#9 MeSH descriptor: [Outcome] explode all trees

# 10 MeSH descriptor: [Mortality] explode all trees
# 11 MeSH descriptor: [Hospital stay] explode all trees
#12 (#1 or #2)
#13 (#3 or #4 or #5)
#14 (#6 or #7 or #8 or #9 or #10 or #11)
#15 (#12 and #13 and #14

## Supplement Checklist. PRISMA checklist

| Section and Topic | Item # | Checklist item | Location where item is reported |
|---|---|---|---|
| **TITLE** | | | |
| Title | 1 | Identify the report as a systematic review. | Page 1 |
| **ABSTRACT** | | | |
| Abstract | 2 | See the PRISMA 2020 for Abstracts checklist. | Page 1 |
| **INTRODUCTION** | | | |
| Rationale | 3 | Describe the rationale for the review in the context of existing knowledge. | Page 2 |
| Objectives | 4 | Provide an explicit statement of the objective(s) or question(s) the review addresses. | Page 2 |
| **METHODS** | | | |
| Eligibility criteria | 5 | Specify the inclusion and exclusion criteria for the review and how studies were grouped for the syntheses. | Page 2 |
| Information sources | 6 | Specify all databases, registers, websites, organisations, reference lists and other sources searched or consulted to identify studies. Specify the date when each source was last searched or consulted. | Page 2 |
| Search strategy | 7 | Present the full search strategies for all databases, registers and websites, including any filters and limits used. | Page 2 |
| Selection process | 8 | Specify the methods used to decide whether a study met the inclusion criteria of the review, including how many reviewers screened each record and each report retrieved, whether they worked independently, and if applicable, details of automation tools used in the process. | Page 2-3 |
| Data collection process | 9 | Specify the methods used to collect data from reports, including how many reviewers collected data from each report, whether they worked independently, any processes for obtaining or confirming data from study investigators, and if applicable, details of automation tools used in the process. | Page 2-3 |
| Data items | 10a | List and define all outcomes for which data were sought. Specify whether all results that were compatible with each outcome domain in each study were sought (e.g. for all measures, time points, analyses), and if not, the methods used to decide which results to collect. | Page 2-3 & Table 2 |
| | 10b | List and define all other variables for which data were sought (e.g. participant and intervention characteristics, funding sources). Describe any assumptions made about any missing or unclear information. | Page 2-3 & Table 1 |
| Study risk of bias assessment | 11 | Specify the methods used to assess risk of bias in the included studies, including details of the tool(s) used, how many reviewers assessed each study and whether they worked independently, and if applicable, details of automation tools used in the process. | Page 2-3 |
| Effect measures | 12 | Specify for each outcome the effect measure(s) (e.g. risk ratio, mean difference) used in the synthesis or presentation of results. | Page 2-3 |
| Synthesis methods | 13a | Describe the processes used to decide which studies were eligible for each synthesis (e.g. tabulating the study intervention characteristics and comparing against the planned groups for each synthesis (item #5)). | Page 3 Figure 1 |
| | 13b | Describe any methods required to prepare the data for presentation or synthesis, such as handling of missing summary statistics, or data conversions. | Page 3 Figures 1 |
| | 13c | Describe any methods used to tabulate or visually display results of individual studies and syntheses. | Table 1 |
| | 13d | Describe any methods used to synthesize results and provide a rationale for the choice(s). If meta-analysis was performed, describe the model(s), method(s) to identify the presence and extent of statistical heterogeneity, and software package(s) used. | Page 3 |

| Section and Topic | Item # | Checklist item | Location where item is reported |
|---|---|---|---|
| | 13e | Describe any methods used to explore possible causes of heterogeneity among study results (e.g. subgroup analysis, meta-regression). | Page 3 |
| | 13f | Describe any sensitivity analyses conducted to assess robustness of the synthesized results. | Page 3 |
| Reporting bias assessment | 14 | Describe any methods used to assess risk of bias due to missing results in a synthesis (arising from reporting biases). | Page 3 |
| Certainty assessment | 15 | Describe any methods used to assess certainty (or confidence) in the body of evidence for an outcome. | Page 3 |
| **RESULTS** | | | |
| Study selection | 16a | Describe the results of the search and selection process, from the number of records identified in the search to the number of studies included in the review, ideally using a flow diagram. | Page 3 & Figures 1 |
| | 16b | Cite studies that might appear to meet the inclusion criteria, but which were excluded, and explain why they were excluded. | Page 3 & Figures 1 |
| Study characteristics | 17 | Cite each included study and present its characteristics. | Page 3 & Table 1 |
| Risk of bias in studies | 18 | Present assessments of risk of bias for each included study. | Page 5-6 Supplemental figure 6 & 7 |
| Results of individual studies | 19 | For all outcomes, present, for each study: (a) summary statistics for each group (where appropriate) and (b) an effect estimate and its precision (e.g. confidence/credible interval), ideally using structured tables or plots. | Page 3-6 Figure 2-5 |
| Results of syntheses | 20a | For each synthesis, briefly summarise the characteristics and risk of bias among contributing studies. | Page 5-6 Supplemental figure 6 & 7 |
| | 20b | Present results of all statistical syntheses conducted. If meta-analysis was done, present for each the summary estimate and its precision (e.g. confidence/credible interval) and measures of statistical heterogeneity. If comparing groups, describe the direction of the effect. | Page 3-6 Figure 2-5 Supplemental figure 1-5 |
| | 20c | Present results of all investigations of possible causes of heterogeneity among study results. | Page 5-6 Supplemental figure 6 & 7 |
| | 20d | Present results of all sensitivity analyses conducted to assess the robustness of the synthesized results. | Page 3-6 |
| Reporting biases | 21 | Present assessments of risk of bias due to missing results (arising from reporting biases) for each synthesis assessed. | Page 5-6 Supplemental figure 6-7 |
| Certainty of evidence | 22 | Present assessments of certainty (or confidence) in the body of evidence for each outcome assessed. | Page 6 Supplemental table 1-2 |
| **DISCUSSION** | | | |
| Discussion | 23a | Provide a general interpretation of the results in the context of other evidence. | Page 6-10 |
| | 23b | Discuss any limitations of the evidence included in the review. | Page 10 |
| | 23c | Discuss any limitations of the review processes used. | Page 10 |
| | 23d | Discuss implications of the results for practice, policy, and future research. | Page 10 |
| **OTHER INFORMATION** | | | |
| Registration and protocol | 24a | Provide registration information for the review, including register name and registration number, or state that the review was not registered. | Page 2 Supplement text 2 |
| | 24b | Indicate where the review protocol can be accessed, or state that a protocol was not prepared. | Page 2 Supplement text 2 |
| | 24c | Describe and explain any amendments to information provided at registration or in the protocol. | Page 2 Supplement text 2 |

| Section and Topic | Item # | Checklist item | Location where item is reported |
|---|---|---|---|
| Support | 25 | Describe sources of financial or non-financial support for the review, and the role of the funders or sponsors in the review. | Page 11 |
| Competing interests | 26 | Declare any competing interests of review authors. | Page 11 |
| Availability of data, code and other materials | 27 | Report which of the following are publicly available and where they can be found: template data collection forms; data extracted from included studies; data used for all analyses; analytic code; any other materials used in the review. | Page 11-12 |

# Supplement Text 2. PROSPERO protocol registration

1. * Review title

Comparison with COVID-19 versus influenza on the outcomes of acute kidney injury: A systematic review and Meta-analysis

2. *Original language title.

Comparison with COVID-19 versus influenza on the outcomes of acute kidney injury: A systematic review and Meta-analysis

3. * Anticipated or actual start date.

15/02/2022

4. * Anticipated completion date.

15/05/2022

5. * Stage of review at time of this submission.

The review has not yet started: No

**PROSPERO**

**International prospective register of systematic reviews**

| Review stage | Started | Completed |
|---|---|---|
| Preliminary searches | Yes | No |
| Piloting of the study selection process | Yes | No |
| Formal screening of search results against eligibility criteria | Yes | No |
| Data extraction | No | No |
| Risk of bias (quality) assessment | Yes | No |
| Data analysis | Yes | No |

Provide any other relevant information about the stage of the review here.

6. * Named contact.

Chiu-Ying Hsiao

Email salutation (e.g. "Dr Smith" or "Joanne") for correspondence:

Mrs Hsiao

1. * Named contact email.

hsiao0101@gmail.com

2. *Named contact address

No. 33, Ln. 300, Wenxian 1st Rd., North Dist., Tainan City, 704011 , Taiwan (R.O.C.)

9. Named contact phone number.

+886-912686027

10. * Organisational affiliation of the review.

Department of Internal Medicine, Chi Mei Medical Center, Tainan, Taiwan

Organisation web address:

http://sub.chimei.org.tw/57300/

11. * Review team members and their organisational affiliations.

Mrs Chiu-Ying Hsiao. Department of Internal Medicine, Chi Mei Medical Center, Tainan, Taiwan
Mr Jui-Yi Chen. Department of Internal Medicine, Chi Mei Medical Center, Tainan, Taiwan

## 12. * Funding sources/sponsors.
nil

## Grant number(s)
nil

## 13. * Conflicts of interest.
None

## 14. Collaborators.
Mr Jui-Yi Chen. Division of Nephrology, Department of Internal Medicine, Chi Mei Medical Center, Tainan, Taiwan

## 15. * Review question.
What are the differences in outcomes of acute kidney injury in patients with SARS-CoV-2 as compared with influenza?

## 16. * Searches.
PubMed, Embase and Cochrane

## 17. URL to search strategy.
Do not make this file publicly available until the review is complete

## 18. * Condition or domain being studied.
Acute kidney injury, COVID-19, influenza

## 19. * Participants/population.
hospitalized adults (18y/o) with COVID-19 or influenza, excluding ESRD with renal replacement therapy

## 20. * Intervention(s), exposure(s).
A group of hospital adult in-patients who were exposed to COVID-19

## 21. * Comparator(s)/control.
A group of hospital adult in-patients who were exposed to influenza

## 22. * Types of study to be included.
We will included retrospective case-control cohort studies, which compared the clinical characteristics, comorbidities and clinical outcomes, including acute kidney injury (AKI), between influenza and COVID-19 infection.

## 23. Context.
Studies which had compared the clinical outcomes including acute kidney injury (AKI) between influenza and COVID-19 infection will be enrolled.

## 24. * Main outcome(s).
The incidence of acute kidney injury, renal replacement therapy (RRT), recovery from AKI, the in-hospital mortality with AKI

## Measures of effect
The effect size is expressed as the pooled odds ratio and 95% Confidence interval

## 26. * Data extraction (selection and coding).
The following data were extracted from the full-text articles: the first author name, year of publication, sample size, study design, patient inclusion criteria, patient demographics, comorbidities, and clinical outcome

## 27. * Risk of bias (quality) assessment.
Newcastle-Ottawa Scale Quality Assessment of included studies to evaluate the bias of studies

## 28. * Strategy for data synthesis..

The odds ratio for outcomes of AKI in COVID-19 and influenza groups will be compared using random effect meta-analysis. Funnel plots and Egger's test were used to examine potential publication bias. Between-trial heterogeneity was determined by using $I^2$ tests and values 50% were regarded as considerable heterogeneity. Statistical significance was defined as p-values 0.05, except for the determination of publication bias that employed p 0.10. We used STATA (Version 16, Stata Corp. 2019, College Station, TX, Stata Corp LP) for all statistical analyses

## 29. * Analysis of subgroups or subsets.

We performed subgroup analysis for including age, sex, participants number, and outcomes, which included the incidence of AKI, renal replacement therapy (RRT), recovery from AKI, the in-hospital mortality with AKI, all-cause mortality and other complications, such as, admission rate to ICU, ventilator therapy, ECMO

## 30. * Type and method of review.

### Type of review

Cost effectiveness
No
Diagnostic
No
Epidemiologic
No
Individual patient data (IPD) meta-analysis
Yes
Intervention
No
Living systematic review
No
Meta-analysis
Yes
Methodology
No
Narrative synthesis
No
Network meta-analysis
No
Pre-clinical
No
Prevention
No
Prognostic
No
Prospective meta-analysis (PMA)
No
Review of reviews
No
Service delivery
No
Synthesis of qualitative studies

No
Systematic review
Yes
Other
No

### Health area of the review

Alcohol/substance misuse/abuse
No
Blood and immune system
No
Cancer
No
Cardiovascular

No
Care of the elderly
No
Child health
No
Complementary therapies
No
COVID-19
Yes
For COVID-19 registrations please tick all categories that apply. Doing so will enable your record to appear in area-specific searches
Chinese medicine
Diagnosis
Epidemiological
Genetics
Health impacts
Immunity
Long COVID
Mental health
PPE
Prognosis
Public health intervention
Rehabilitation
Service delivery
Transmission
Treatments
Vaccines
Other
Crime and justice
No
Dental
No
Digestive system
No
Ear, nose and throat
No
Education
No
Endocrine and metabolic disorders
No
Eye disorders
No
General interest
No
Genetics
No
Health inequalities/health equity
No
Infections and infestations
No
International development
No
Mental health and behavioural conditions
No
Musculoskeletal
No
Neurological
No
Nursing
No
Obstetrics and gynaecology
No
Oral health
No

Palliative care
No
Perioperative care
No
Physiotherapy
No
Pregnancy and childbirth
No
Public health (including social determinants of health)
No
Rehabilitation
No
Respiratory disorders
No
Service delivery
No
Skin disorders
No
Social care
No
Surgery
No
Tropical Medicine
No
Urological
No
Wounds, injuries and accidents
No
Violence and abuse
No

## 31. Language.

English
There is not an English language summary

## 32. * Country.

Taiwan

## 33. Other registration details.

none

## 34. Reference and/or URL for published protocol.

No I do not make this file publicly available until the review is complete

## 35. Dissemination plans.

No

## 36. Keywords.

Acute kidney injury, COVID-19, influenza, outcome

## 37. Details of any existing review of the same topic by the same authors.

none

## 38. * Current review status.

Review_Ongoing

## 39. Any additional information.

none

## 40. Details of final report/publication(s) or preprints if available.

none

## Supplement Figures

**Supplement Figure 1. Forest plot depicted subgroup analysis of influenza type (influenza A, influenza A or B), age ( <65 years old or ≥65 years old), sex (female, <50%, ≥50%), the proportion of ICU admission (<100%, 100%), shock (<50%, ≥50%) and using vasopressor(%) for the risk of acute kidney injury**

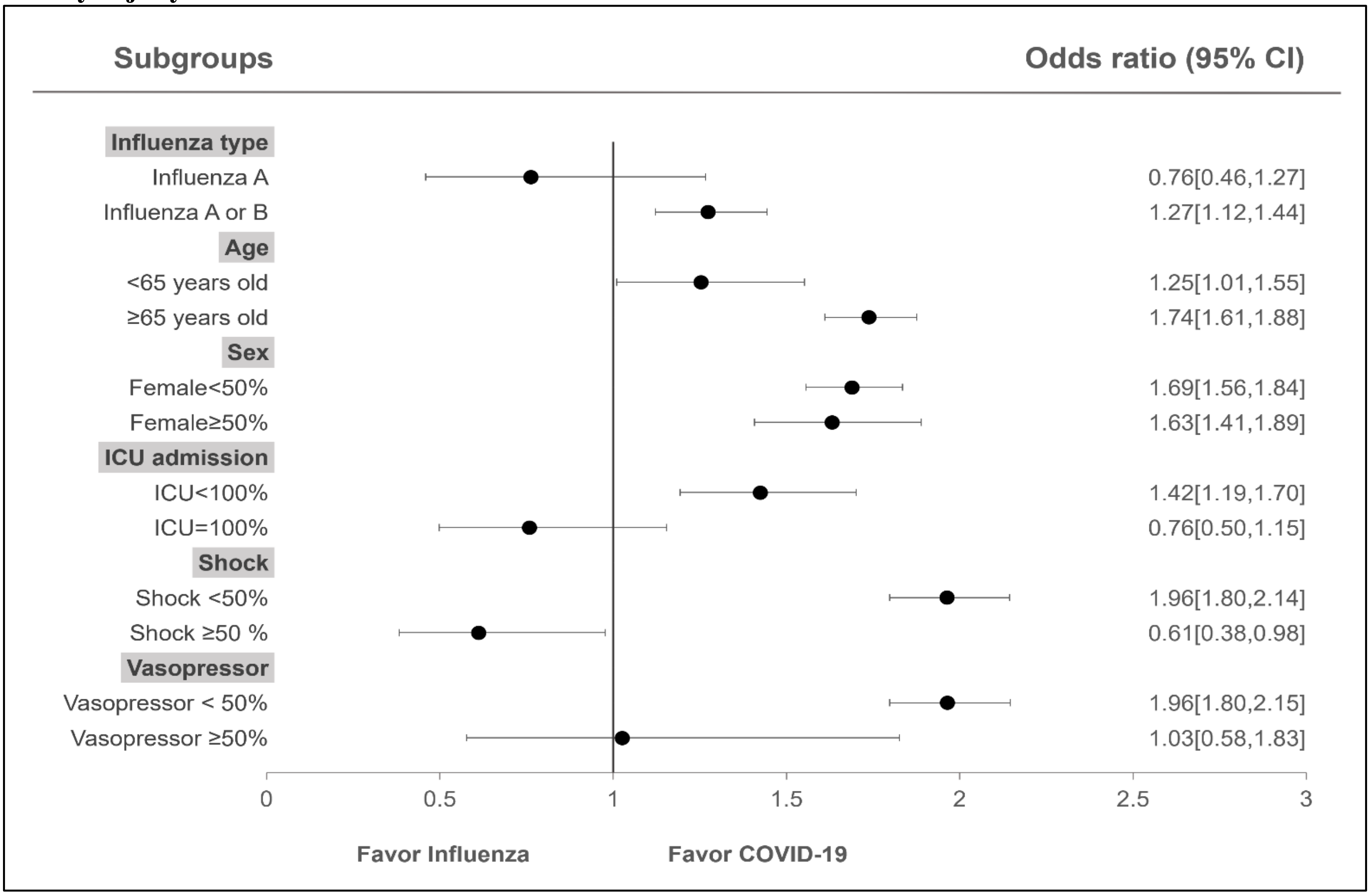


Abbreviations: CI, Confidence Interval; COVID-19, Coronavirus Disease 2019; ICU, Intensive Care Unit

**Supplement Figure 2. Forest plot depicted the risk of renal replacement therapy between COVID-19 and influenza patients**

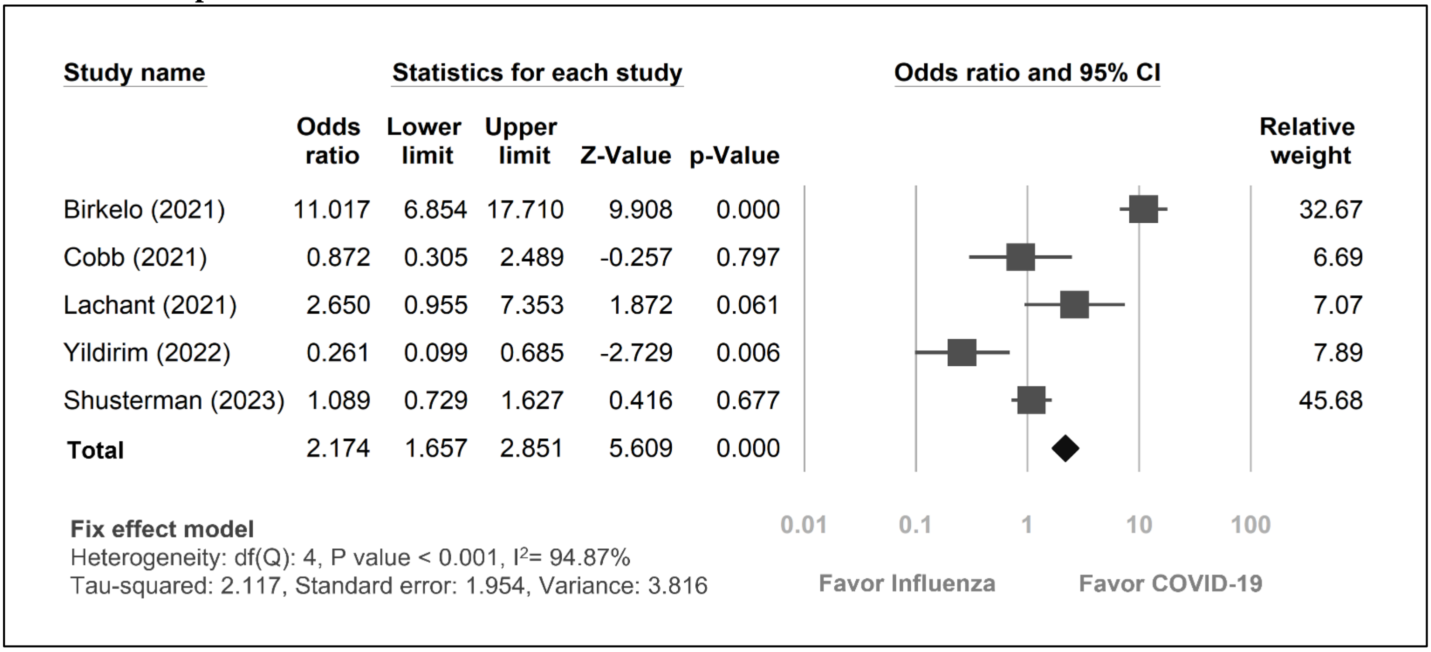


Abbreviations: CI, Confidence Interval; COVID-19, Coronavirus Disease 2019; ICU, Intensive Care Unit

**Supplement Figure 3. Forest plot depicted the risk of Vasopressor therapy between COVID-19 and influenza patients**

| Study name | Odds ratio | Lower limit | Upper limit | Z-Value | p-Value | Relative weight |
|---|---|---|---|---|---|---|
| Bhasin (2021) | 1.576 | 1.007 | 2.467 | 1.991 | 0.047 | 16.71 |
| Birkelo (2021) | 8.974 | 6.808 | 11.830 | 15.569 | 0.000 | 43.97 |
| Cobb (2021) | 1.310 | 0.671 | 2.557 | 0.792 | 0.428 | 7.51 |
| Cousin (2021) | 0.375 | 0.115 | 1.220 | -1.629 | 0.103 | 2.41 |
| Lachant (2021) | 5.480 | 3.108 | 9.661 | 5.880 | 0.000 | 10.44 |
| Nasir (2021) | 0.560 | 0.250 | 1.257 | -1.405 | 0.160 | 5.15 |
| Zheng (2021) | 1.331 | 0.813 | 2.179 | 1.138 | 0.255 | 13.81 |
| **Total** | 3.404 | 2.834 | 4.088 | 13.105 | 0.000 | |

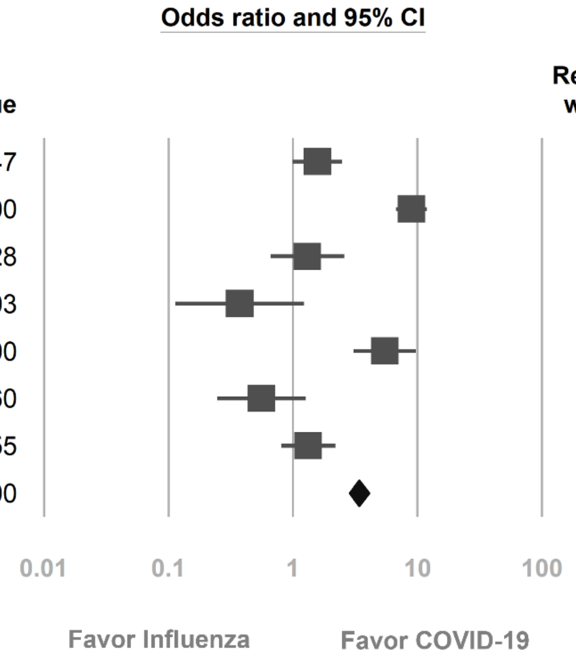


**Fix effect model**
Heterogeneity: df(Q): 6, P value< 0.001, $I^2$= 94.82%
Tau-squared: 1.295, Standard error: 0.959, Variance: 0.919

Abbreviations: CI, Confidence Interval; COVID-19, Coronavirus Disease 2019; ICU, Intensive Care Unit

**Supplement Figure 4. Forest plot depicted the risk of ventilator therapy between COVID-19 and influenza patients**

| Study name | Odds ratio | Lower limit | Upper limit | Z-Value | p-Value | Relative weight |
|---|---|---|---|---|---|---|
| Bhasin_2021 | 2.147 | 1.300 | 3.545 | 2.984 | 0.003 | 6.52 |
| Birkelo_2021 | 6.340 | 5.150 | 7.804 | 17.420 | 0.000 | 37.99 |
| Cobb_2021 | 0.091 | 0.030 | 0.282 | -4.169 | 0.000 | 1.30 |
| Cousin_2021 | 4.256 | 0.165 | 109.523 | 0.874 | 0.382 | 0.16 |
| Lachant_2021 | 4.675 | 2.858 | 7.645 | 6.144 | 0.000 | 6.78 |
| Nasir_2021 | 0.321 | 0.162 | 0.634 | -3.273 | 0.001 | 3.53 |
| Tang_2020 | 0.064 | 0.029 | 0.144 | -6.697 | 0.000 | 2.54 |
| Zheng_2021 | 1.648 | 0.599 | 4.534 | 0.968 | 0.333 | 1.60 |
| Yildirim_2022 | 0.392 | 0.180 | 0.850 | -2.373 | 0.018 | 2.73 |
| Shusterman_2023 | 2.034 | 1.647 | 2.512 | 6.596 | 0.000 | 36.85 |
| **Total** | 2.615 | 2.300 | 2.972 | 14.709 | 0.000 | |

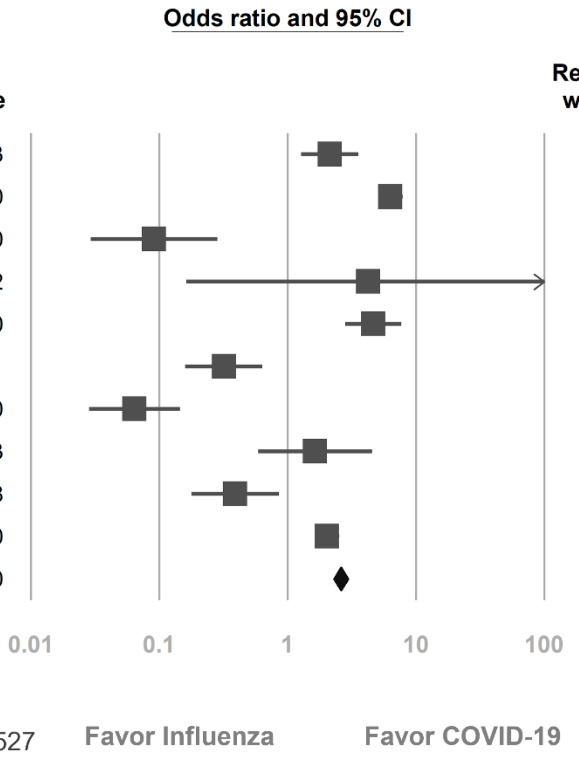


**Fix effect model**
Heterogeneity: df(Q): 9, P value< 0.001, $I^2$= 96.51%
Tau-squared: 1.499, Standard error: 1.236, Variance: 1.527

Abbreviations: CI, Confidence Interval; COVID-19, Coronavirus Disease 2019; ICU, Intensive Care Unit

**Supplement Figure 5. Forest plot depicted the risk of acute respiratory distress syndrome between COVID-19 and influenza patients**

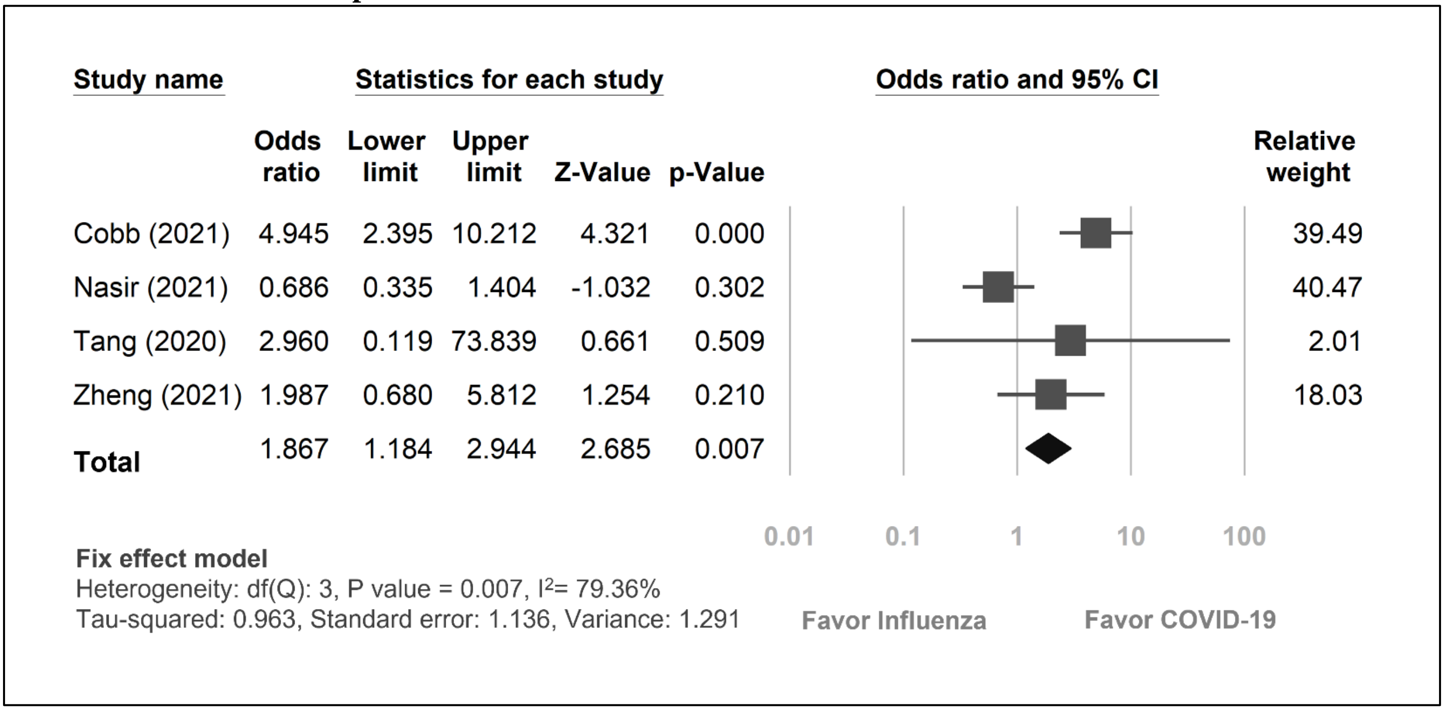


Abbreviations: CI, Confidence Interval; COVID-19, Coronavirus Disease 2019; ICU, Intensive Care Unit

**Supplement Figure 6. Funnel plot depicted the risk of acute kidney injury between COVID-19 and influenza patients**

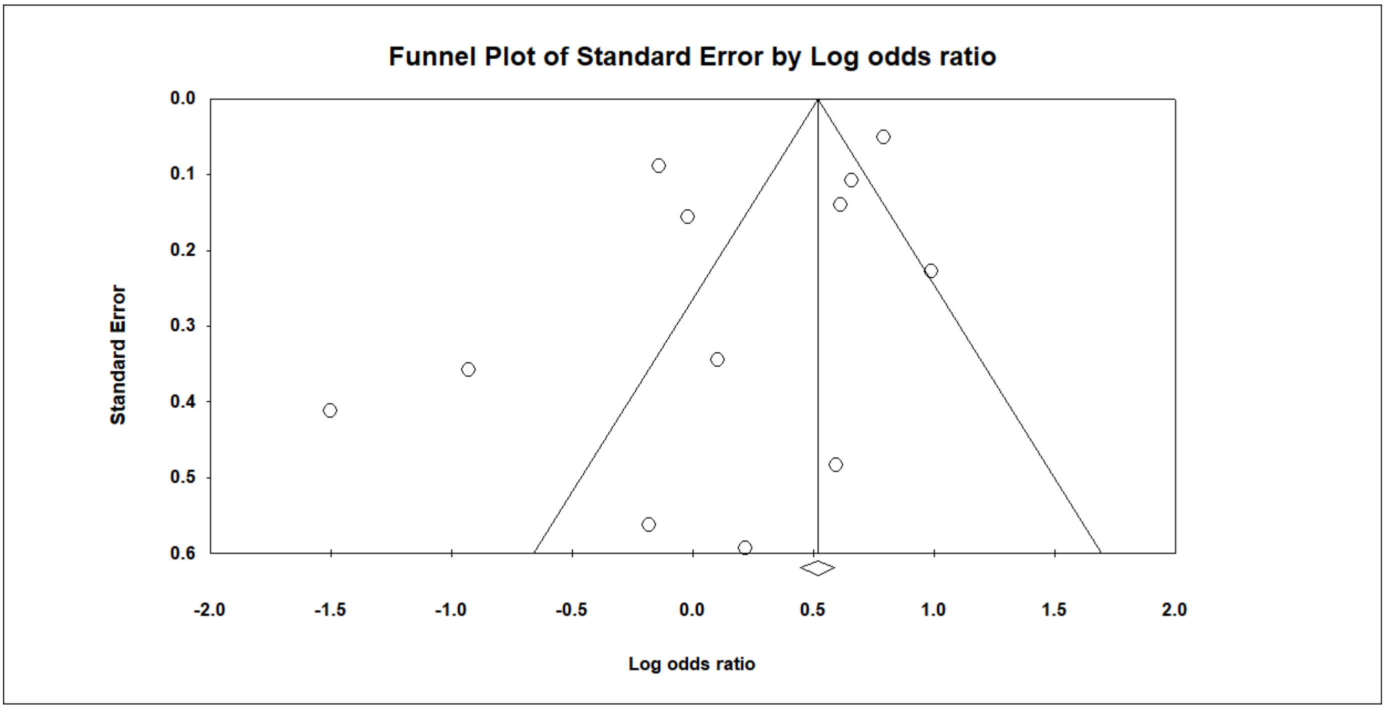

**Supplement Figure 7 The meta-regression bubble plot showing the effect modification of (A)age (B)DM (C)Hypertension (D) CKD and the risk of acute kidney injury according to COVID-19 and influenza infection.**

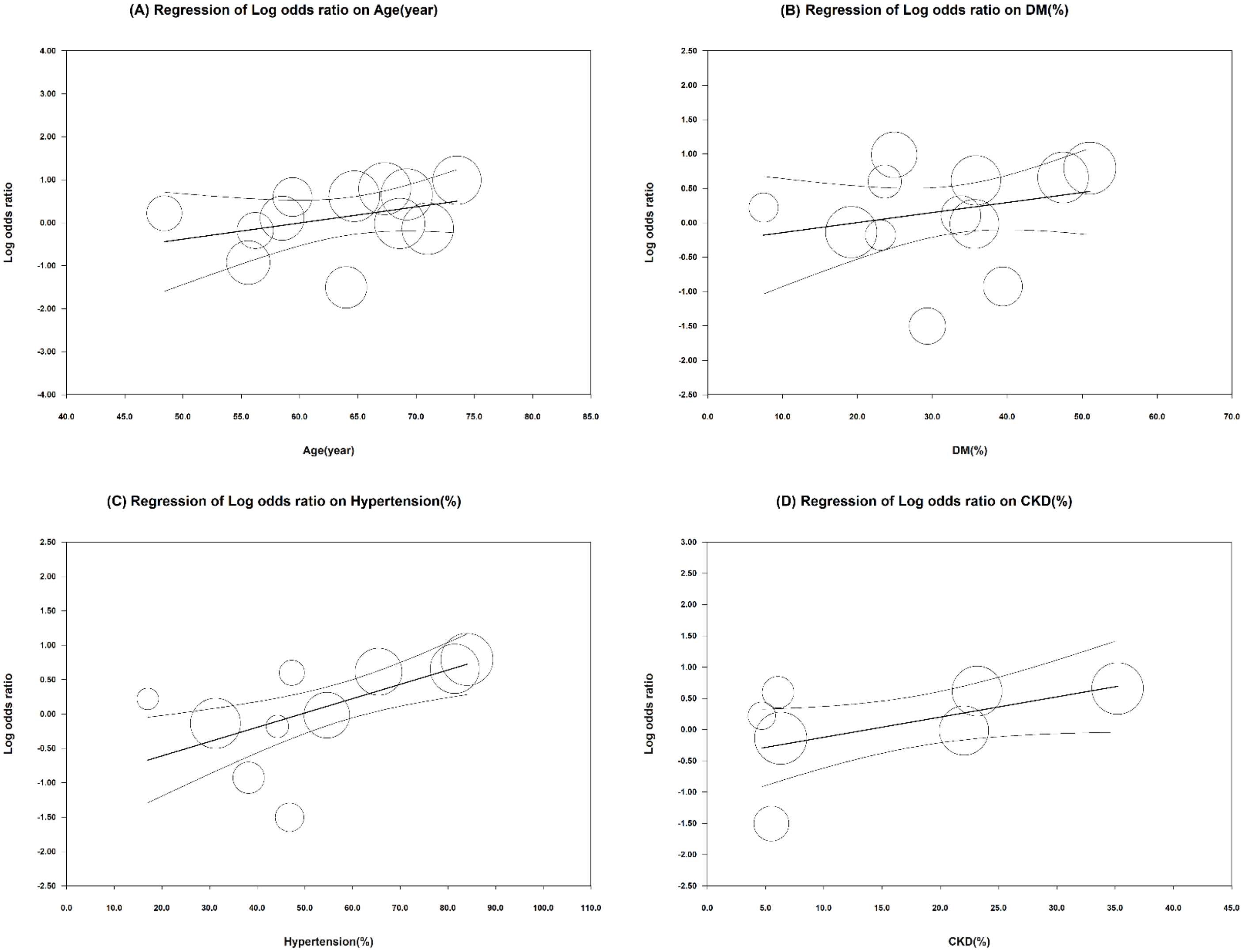


Abbreviations: CKD, chronic kidney disease; COVID-19, Coronavirus Disease 2019; DM, Diabetes Mellitus

**Supplementary Table 1. Newcastle-Ottawa Scale Quality Assessment of included studies**

| First author _ Year | Selection | | | | Comparability | Exposure | | | Total |
|---|---|---|---|---|---|---|---|---|---|
| | Representativeness of the exposed cohort | Selection of the non exposed cohort | Ascertainment of exposure | Demonstration that outcome of interest was not present at start of study | Comparability of cohorts on the basis of the design or analysis | Assessment of outcome | Was follow-up long enough for outcomes to occur | Adequacy of follow up of cohorts | |
| Bhasin_2021 | * | * | * | * | ** | * | * | * | 9 |
| Birkelo_2021 | * | * | * | * | ** | * | * | * | 9 |
| Cobb_2021 | * | * | * | * | * | * | * | * | 8 |
| Cousin_2021 | * | * | * | * | * | * | * | * | 8 |
| Erich_2021 | * | * | * | * | * | * | * | * | 8 |
| Lachant_2021 | * | * | * | * | * | * | * | * | 8 |
| Nasir_2021 | * | * | * | * | * | * | * | * | 8 |
| Strohbehn_2021 | * | * | * | * | ** | * | * | * | 9 |
| Tang_2020 | * | - | * | * | * | * | * | * | 7 |
| Zheng_2021 | * | * | * | * | * | * | * | * | 8 |
| Yildirim_2022 | * | * | * | * | * | * | * | * | 8 |
| Shusterman_2023 | * | * | * | * | * | * | * | * | 8 |

**Supplement Table 2. Quality assessment the GRADE results**

**Summary of findings:**

**COVID-19 compared to Influenza for AKI**

**Patient or population:** AKI
**Setting:**
**Intervention:** COVID-19
**Comparison:** Influenza

| Outcomes | Anticipated absolute effects* (95% CI) | | Relative effect (95% CI) | № of participants (studies) | Certainty of the evidence (GRADE) | Comments |
|---|---|---|---|---|---|---|
| | Risk with Influenza | Risk with COVID-19 | | | | |
| Incidence of AKI | 210 per 1,000 | **308 per 1,000** (293 to 323) | **OR 1.675** (1.559 to 1.800) | 17618 (12 observational studies) | ⊕⊕◯◯ Low | The evidence suggests that COVID-19 infection had higher risk of incidence of AKI compared with Influenza infection. |
| In-hospital mortality | 55 per 1,000 | **256 per 1,000** (126 to 451) | **OR 5.90** (2.47 to 14.12) | 3000 (3 observational studies) | ⊕⊕⊕◯ Moderate | The evidence suggests that COVID-19 infection had higher risk of in-hospital mortality compared with Influenza infection. |
| Recovery from AKI | 802 per 1,000 | **607 per 1,000** (460 to 737) | **OR 0.381** (0.210 to 0.692) | 2297 (2 observational studies) | ⊕⊕◯◯ Low | The evidence suggests that Influenza infection had higher possibility of recovery from AKI compared with COVID-19 infection. |

***The risk in the intervention group** (and its 95% confidence interval) is based on the assumed risk in the comparison group and the **relative effect** of the intervention (and its 95% CI).

**CI:** confidence interval; **OR:** odds ratio

**GRADE Working Group grades of evidence**
**High certainty:** we are very confident that the true effect lies close to that of the estimate of the effect.
**Moderate certainty:** we are moderately confident in the effect estimate: the true effect is likely to be close to the estimate of the effect, but there is a possibility that it is substantially different.
**Low certainty:** our confidence in the effect estimate is limited: the true effect may be substantially different from the estimate of the effect.
**Very low certainty:** we have very little confidence in the effect estimate: the true effect is likely to be substantially different from the estimate of effect.
